# Greater fatigue is more strongly associated with reduced reward sensitivity in the long-term phase of coronavirus disease (COVID-19) than in the early phase

**DOI:** 10.1016/j.bbih.2025.101056

**Published:** 2025-07-05

**Authors:** Judith M. Scholing, Britt I.H.M. Lambregts, Ruben van den Bosch, Esther Aarts, Marieke E. van der Schaaf

**Affiliations:** a Centre for Cognitive Neuroimaging, Donders Institute for Brain, Cognition and Behaviour, Radboud University, Nijmegen, the Netherlands; b Department of Psychiatry, Radboud University Medical Center, Nijmegen, the Netherlands; c Tilburg University, Department of Cognitive Neuropsychology, Tilburg, the Netherlands

**Keywords:** COVID-19, Post-COVID-19, Chronic fatigue, Motivation, Effort-based decision making

## Abstract

**Background:**

Fatigue and depressive mood is inherent to acute disease, but a substantial group of people report persisting disabling fatigue and depressive symptoms long after a COVID-19 infection. Infections have been shown to change decisions about engaging in effortful and rewarding activities, but it is currently unclear whether fatigue and depressive symptoms are similarly associated with decision making during early and persistent phases after a COVID-19 infection. Here, we investigated whether fatigue and depressive mood are associated with altered weighting of reward and effort in decision making at different timepoints after COVID-19 infection.

**Methods:**

We conducted an online cross-sectional study between March 2021 and March 2022, in which 242 participants (18–65 years) with COVID-19 < 4 weeks ago (n = 62), COVID-19 > 12 weeks ago (n = 81), or no prior COVID-19 (n = 90; self-reported) performed an effort-based decision-making task. In this task, participants accepted or rejected offers in which they could exert physical effort (ticking boxes on screen, 5 levels) to gain rewards (money to be gained in a voucher-lottery, 5 levels). State fatigue and depressive mood were measured with the Profile of Mood States (POMS) prior to the task. We used mixed binomial regression analysis to test whether fatigue and depressive mood were related to acceptance rates for reward and effort levels and whether this differed between the groups.

**Results:**

Compared with no COVID-19 and COVID-19 < 4 weeks groups, the COVID-19 > 12 weeks group reported higher state fatigue (mean ± SD: 20 ± 7 vs. 14 ± 7 and 12 ± 6 POMS-score, respectively; both p < 0.001) and was less sensitive to rewards (Reward∗Group: OR: 0.35 (95 %CI 0.20, 0.62), p < 0.001 and OR: 0.38 (95 %CI 0.20, 0.72), p = 0.003). In the COVID-19 > 12 weeks group, fatigue was more negatively associated with reward sensitivity compared to the COVID-19 < 4 weeks group (Reward∗Fatigue∗Group: OR 0.47 (95 %CI 0.25, 1.13), p = 0.022) and the no COVID-19 group (OR 0.48 (95 %CI 4.01, 0.92), p = 0.029). No group differences were observed for the relationship between fatigue and effort sensitivity. There were also no group differences for the relationship between depressive mood and effort or reward sensitivity. Higher age, lower BMI, unhealthy lifestyle, and worrying during the early phase of COVID-19 each predicted lower reward sensitivity in the > 12 weeks group (Age∗Reward: OR 0.30 (95 %CI 0.19, 0.48), p < 0.001; BMI∗Reward: OR 1.43 (95 %CI 1.01, 2.00), p = 0.047); Lifestyle∗Reward: OR 1.50 (95 %CI 1.06, 2.14), p = 0.022; Worrying∗Reward: OR 0.59 (95 %CI 0.38, 0.94), p = 0.025, respectively).

**Conclusion:**

The finding that fatigue is related to lower reward sensitivity > 12 weeks after COVID-19 suggests potential reward deficits in post-COVID-19 fatigue. Moreover, higher age, unhealthy lifestyle, and worrying during the early phase of COVID-19 are potential risk factors for developing lower reward sensitivity. These findings are in line with previous observations that long-term inflammation induces dysregulations in neural reward processing, which should be investigated in future studies.

## Highlights

• We tested how fatigue and mood relate to decision making after COVID-19 or no COVID.
• Fatigue was greater and reward sensitivity smaller >12wk than <4wk post-COVID.
• Fatigue was more related to lower reward sensitivity in >12wk than <4wk post-COVID.
• Depressive mood >12wk post-COVID was not associated with altered decision making.
• Older age, unhealthy lifestyle, and worrying predicted lower reward sensitivity.

## **Abbreviations**:

•ANOVA: Analysis of variance
•COVID-19: Coronavirus disease 2019
•BMI: Body mass index
•ISCED: International Standard Classification of Education
•LPS: Lipopolysaccharide
•MBT: Maximum number of boxes ticked
•MFI: Multidimensional Fatigue Index
•POMS: Profile of Mood States
•SARS-CoV-2: Severe respiratory syndrome coronavirus 2
•SF-36: 36-Item Short Form Health Survey
•95 % CI: 95 % confidence interval

## 1 Introduction

Flu-like symptoms such as fever, coughing, fatigue and depressed mood are common during an infection with severe respiratory syndrome coronavirus 2 (SARS-CoV-2) or coronavirus disease 2019 (COVID-19). However, a substantial number of people report persistence of symptoms at least three months after infection with SARS-CoV-2, which has been defined by the World Health Organisation as post-COVID-19 syndrome (Soriano et al., 2022). Patients report a wide variety of physical and mood-related symptoms (Lopez-Leon et al., 2021), with fatigue and depression being the most commonly reported, but still poorly understood, symptoms.

These symptoms considerably impact daily and occupational functioning. While fatigue is a feeling of physical or mental exhaustion that promotes rest-taking behaviour, depression is characterized by a reduced ability to experience pleasure (Cooper et al., 2018; Swardfager et al., 2016). Both fatigue and depression affect daily functioning by changing decision making. Specifically, they have been associated with alterations in decisions to engage in activities that involve weighing of the costs (e.g. effort investments) against the benefits (e.g. the experience of pleasure) of that activity (Erfanian Abdoust et al., 2024; Matthews et al., 2023; Treadway et al., 2012, p. 202). However, how fatigue and depression relate to decision making and whether this is the same during early and persistent phases after a COVID-19 infection is currently unclear. Assessing the behavioural characteristics of fatigue and depression by means of decision making could therefore help to better understand the differential impact of these symptoms on daily and occupational functioning during early and chronic phases of COVID-19 infections.

During a microbial or viral infection, systemic inflammation induces a process known as sickness behaviour, which involves feelings of fatigue and depression, but also increases in rest-taking behaviours (Dantzer, 2001a). Sickness behaviour is considered to be an adaptive behavioural change that promotes recovery by directing bodily energy resources towards internal processes that fight disease and away from (effortful) physical or mental activities (Dantzer, 2001b). Previous research has shown that inflammation induced by lipopolysaccharide (LPS) administration or cancer treatment is associated with alterations in motivational behaviour (De Marco et al., 2023; Lacourt et al., 2018; Lasselin et al., 2017). In contrast, studies investigating inflammation induced by influenza vaccination or acute psychosocial stress did not report such effects (Boyle et al., 2019, 2020). These contradictory findings may partly be explained by methodological differences, particularly the inability to disentangle the role of effort and reward sensitivity. To address this, studies in the field of cognitive neuroscience have investigated sickness behaviours using effort-based decision-making tasks (Draper et al., 2018; Lambregts et al., 2023; Matthews et al., 2023; Müller and Apps, 2019). In this type of decision-making tasks, one decides whether or not to perform an effortful task by weighing the amount of effort it will cost against the reward it will bring (Chong et al., 2016). By presenting multiple offers with various combination of reward and effort levels, it is possible to analyse choice-behaviour to derive individual parameters of effort- and reward-sensitivity. These parameters indicate how reward and effort information influence decisions to engage in activities. Specifically, effort sensitivity reflects the extent to which increasing effort influences the decision to engage in an activity, while reward sensitivity reflects the extent to which the rewarding outcome affects the decision to engage. Previous research using this type of tasks has shown that acute inflammation induced by bacterial LPS in healthy individuals indeed alters effort-based decision making. Specifically, fatigue during acute systemic inflammation was selectively associated with increased sensitivity to effort but not reward information during decisions to engage in a rewarding but effortful activity (Draper et al., 2018; Lambregts et al., 2023). These results provide insight into which processes are affected by acute inflammation to promote rest-taking behaviours during sickness.

When symptoms after infections persist, their characteristics often change, raising the question whether their relationship to decision making also changes. A study from Capuron and Miller (2011) showed that patients receiving long-lasting treatment with the immune-stimulant interferon-alfa initially only experienced vegetative sickness symptoms, such as fatigue and reduced appetite, in the first 4 weeks. However, after 4–6 weeks, they additionally started to develop mood and cognitive problems, including major depressive disorder (Capuron and Miller, 2011). Similar patterns were observed with COVID-19: Davis et al. (2021) showed that while fatigue (and other general sickness symptoms, e.g. fever) started directly after the infection, other symptoms, including cognitive problems and post-exertional malaise, i.e. worsening of symptoms following physical or mental exertion, started to increase after 4–8 weeks (Davis et al., 2021). This has led to the hypothesis that the mechanism through which inflammation causes acute versus long-term symptoms might be different. For instance, the transition to chronic inflammation has been linked to specific molecular changes, including dysregulation of mesolimbic dopaminergic signaling pathways (Capuron et al., 2012; Felger, 2017; Felger and Lotrich, 2013). Thus, while fatigue during acute inflammation is associated with increased effort sensitivity (Draper et al., 2018; Lambregts et al., 2023), depressive symptoms, which are also common in conditions of chronic low-grade inflammation (Beurel et al., 2020; Kohler et al., 2016; Miller et al., 2009), have been related to decreased sensitivity to reward (Keren et al., 2018; O'Callaghan and Stringaris, 2019; Treadway et al., 2012). This way, both fatigue and depressive mood can result in similar changes in decision making, namely less engagement in effortful – but rewarding – activities. However, the mechanism driving this decision making might be different, and might change during the transition from an acute COVID-19 infection to a post-COVID-19 condition.

**Fig. 1:**
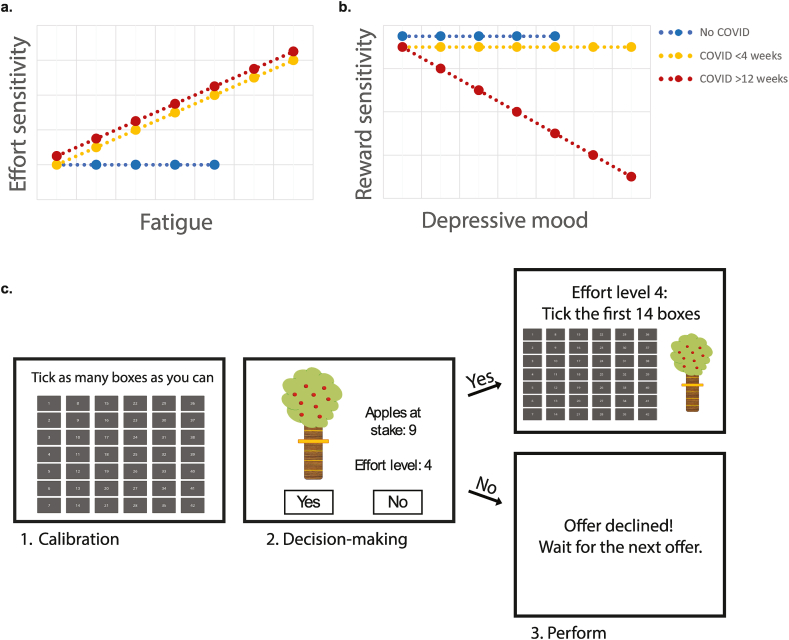
Visual overview of our hypothesis regarding the association between fatigue and effort sensitivity (a), depressive mood and reward sensitivity (b), and the task design (c) of the study.

In this paper, we aimed to investigate how fatigue and depressive mood during different phases after a COVID-19 infection relate to reward and effort sensitivity. We used a well-established computerized effort-based decision-making task (Bonnelle et al., 2016; Contreras-Huerta et al., 2022) to test whether the relationships between reward/effort sensitivity and fatigue/depression symptoms differed between people who had COVID-19 > 12 weeks ago, individuals who had COVID-19 < 4 weeks ago and individuals who did not have COVID-19. Participants with active symptoms as well as resolved symptoms were included in both the < 4 weeks and > 12 weeks groups, enabling us to assess whether higher levels of fatigue and depression in those groups related differently to effort and/or lower reward sensitivity. We hypothesized that fatigue in both the < 4 weeks and > 12 weeks group is more strongly associated with increased effort sensitivity compared to the no COVID-19 group. Additionally, we hypothesize that depressive mood is more strongly associated with decreased reward sensitivity only in the > 12 weeks group compared to the < 4 weeks and no COVID-19 groups (Fig. 1; preregistered at https://osf.io/uhwkc).

Currently, it is unclear why some people develop post-COVID-19 and others do not. To better understand the transition from acute to persistent symptoms, it is important to understand who is at risk for developing post-COVID-19 and possible associated alterations in decision making (Zhao et al., 2023). Previous studies have already identified various factors that predict acute COVID-19 severity, including demographic- and lifestyle-related factors, such as body mass index (BMI), smoking, age and sex (Chudzik et al., 2022; Kofahi et al., 2022; Merino et al., 2021; Pływaczewska-Jakubowska et al., 2022). For post-COVID-19, however, these predictors are less clear: some studies suggest that higher BMI, less exercise and smoking might also increase the risk of developing post-COVID-19 (Bai et al., 2022; Pływaczewska-Jakubowska et al., 2022; Subramanian et al., 2022), while others do not find this association (Crook et al., 2021; Pływaczewska-Jakubowska et al., 2022). One reason for this heterogeneity is that different studies used different ways of measuring post-COVID-19 symptoms with various combinations of physiological, fatigue, and mood-related symptoms. Here, we used the decision-making task to investigate whether we can identify predictors of reward and effort sensitivity in people who had COVID-19 > 12 weeks ago, compared to individuals who had COVID-19 < 4 weeks ago, or did not have COVID-19. In previous cognitive neuroscience studies, similar demographic and lifestyle factors, such as BMI, age, and smoking have indeed been found to be associated with altered decision making (Addicott et al., 2020; Byrne and Ghaiumy Anaraky, 2020; Mansur et al., 2019). By focusing on the independent processes of decision making (i.e. reward and effort sensitivity), we might be able to delineate specific predictors for specific behavioural characteristics of COVID-19.

Taken together, post-COVID symptoms of fatigue and depressive mood are currently poorly understood. This study uses effort-based decision making (1) to investigate how fatigue and depressive mood during different phases after a COVID-19 infection relate to reward and effort sensitivity, and (2) to assess which risk factors predict reward and effort sensitivity in individuals who had COVID-19 > 12 weeks ago. Results of both objectives may provide insight into differential processes that underlie fatigue and depressive symptoms after COVID-19 infections and how this impacts decisions about energy expenditure in daily functioning.

## 2 Materials and methods

### 2.1 Population and design

This online study had a cross-sectional design. We preregistered the study on OSF (available via https://osf.io/uhwkc). In total, we recruited 308 participants through social media (e.g. post-COVID-19 patient groups), flyers and the university's online recruitment database. In- and exclusion criteria were having an age between 18 and 65 years and being able to speak Dutch in order to understand the instructions. Furthermore, we recruited three groups of people: people who had COVID-19 more than 12 weeks ago as counted from the day of symptom onset (n = 113), people who had COVID-19 less than 4 weeks ago as counted from the day of symptom onset (n = 89), and people who never had COVID-19 (self-reported) (n = 106). As our objective was to study the association between fatigue and depressive symptoms with our effort/reward sensitivity measures within these three groups – rather than only comparing these groups on effort/reward measures- we included both participants with symptoms and participants whose symptoms had resolved. We selected the time windows of < 4 weeks and > 12 weeks as these timeframes were used in previous research (Davis et al., 2021; Greenhalgh et al., 2020; Soriano et al., 2022) and were based on the report that inflammatory effects on neuropsychiatric symptoms transition around 8 weeks from predominantly neurovegetative to mood and cognitive symptoms (Capuron and Miller, 2011). Recruitment took place between March 2021 and February 2022 (Fig. S1, Supplementary Material 1).

### 2.2 Procedure

When opening the link of the study, participants first encountered a webpage with information about the study. Participants gave informed consent via an opt-in button, after which they were redirected to the study. Participants were then asked to fill in several questionnaires and perform an online behavioural task, together taking approximately 30 min to complete. Among the participants, we distributed 40 gift cards of 5 euros plus up to 5 euros that participants were able to win during the behavioural task in a lottery draw. The lottery chances were provided to the participants before participating in the study. Participants performed the study in the online questionnaire platform Gorilla Experiment Builder (www.gorilla.sc) (Tomczak et al., 2023), which could only be performed on a computer, laptop, or tablet (not on a smart phone). The study was reviewed by the Medical Ethics Review Committee of Radboudumc in Nijmegen, the Netherlands, which concluded that the study does not fall under the scope of the Dutch Medical Research Involving Human Subjects Act (WMO).

### 2.3 Measurement of fatigue and depression

We measured the main predictors in this study, current state fatigue (scale: 6–30) and depressive mood (scale: 8–40) (i.e. how do you feel right now), by fatigue and depressive mood subscales of the Profile of Mood States (POMS) questionnaire (Grove and Prapavessis, 1992; McNair, 1971) before starting with the experimental task. In addition, for descriptive purposes, we additionally measured overall fatigue in the past two weeks by the Multidimensional Fatigue Inventory (MFI) (scale: 0–100) (Smets et al., 1995).

### 2.4 Experimental task: effort-based decision making

We measured reward and effort sensitivity using an adjusted online version of the decision-making task from Bonnelle et al. (2016) and Contreras-Huerta et al. (2022) (Bonnelle et al., 2016; Contreras-Huerta et al., 2022) (Fig. 1). In this task, participants decided whether a certain reward, i.e. apples in the tree, was worth a certain amount of effort, i.e. clicking a certain number of boxes within 10 s. The task was divided in four parts: calibration, familiarization, decision making and execution. The decision-making and execution phases were separated to avoid that fatigue due to the execution would affect the participant's decisions. The task took approximately 15 min to complete.

During calibration, we measured how many boxes the participant was able to tick with their computer mouse or on their tablet's touch screen within 10 s. This was repeated three times, and the highest number of boxes ticked (maximum number of boxes ticked (MBT)) was used to determine the five levels of effort (i.e. 10, 30, 50, 70, and 90 % of MBT). During familiarization, participants performed all five levels of effort three times to get familiar with each effort level. To validate the experienced task load, participants filled in the NASA Task Load index after each effort level (Hart, 1986).

During the decision phase, we presented participants with offers of combinations consisting of one of 5 levels of effort (10, 30, 50, 70 and 90 % of MBT) with one of 5 levels of reward (1, 3, 6, 9 and 12 apples). Participants had a maximum of 10 s to decide whether they were willing to perform the amount of effort for the reward at stake. All 25 combinations of the reward and effort levels were presented to the participants five times, resulting in a total of 125 trials.

During the execution phase, participants executed 25 trials that were randomly selected from the decision phase. If they had accepted the trial, participants performed the trial and, if successful, collected the apples. Each collected apple corresponded to an additional €0,04 on the €5,- gift card they could win in a lottery. If they had not accepted the trial, participants waited for 10 s until the next trial was presented.

### 2.5 Demographics and population characteristics

We collected information on demographics (including age, sex, education, socio economic status) and population characteristics using existing questionnaires and newly created items. We used the 36-item Short Form Health Survey (Hays et al., 1993) to describe the current physical health status of the population and the MFI to describe the fatigue levels in the past two weeks.

To assess symptoms associated with COVID-19 infection and post-COVID-19, we created a set of 29 physical, cognitive and mood symptoms (fever, shivers, sore throat, coughing, coughing blood, nose cold, loss of smell and/or taste, gastrointestinal problems, rash, headache, muscle pain, chest pain, shortness of breath, heavy limbs, high heart rate, dizziness, eye problems, fatigue, fatigue after mild exertion, long recovery after exertion, concentration problems, confusion, feeling depressed, feeling stressed, feeling anxious, low motivation, sensitive to light, sleep problems, memory loss). We assessed which symptoms participants experienced during the first two weeks of COVID-19 infections on a 5-point scale (1 = not at all, 2 = rarely, 3 = sometimes 4 = often, 5 = always, unvalidated) and we assessed the relative change in symptoms during the two weeks prior to participation as compared to before March 2020 on a 6-point scale (1 = not at all, 2 = much less often, 3 = slightly less often 4 = just as often, 5 = slightly more often, 6 = much more often, unvalidated). An overview of all items and questionnaires are shown in Supplementary Material 2.

### 2.6 Predictors of reward and effort sensitivity

To address our second objective, various items and questionnaires were used to compute the following predictors of reward and effort sensitivity: age (years), sex (male, female), pre-pandemic BMI (kg/m2), pre-pandemic lifestyle, socio-economic status, pre-pandemic physical health, COVID-19 disease severity, worrying during the first two weeks of COVID-19, and use of COVID-19-related aftercare.

For pre-pandemic lifestyle, socio-economic status, pre-pandemic physical health, and COVID-19 disease severity, we calculated a combined score by averaging Z-scores of a set of related variables. Specifically, for lifestyle, we calculated a combined score by averaging Z-scores of pre-pandemic smoking (daily, occasionally, or no smoking), alcohol use (> 4 times a week, 2–3 times a week, 2–4 times a month, monthly, or never), medium and high intensive exercising (never, 1–2h a week, 2–3h a week, 4–5h a week, or > 5h a week), and having diabetes type II (yes, or no), where a higher Z-score for lifestyle score indicates a healthier lifestyle. The questions used for the calculation of the lifestyle score were not validated.

For socio-economic status, we calculated a combined score by averaging Z-scores of income (€ per year, unvalidated) and educational level (ISCED score (International Standard Classification of Education (ISCED), n.d.)).

For pre-pandemic physical health, we calculated a Z-score of the number of pre-pandemic chronic diseases participants had (unvalidated).

For COVID-19 disease severity, we asked participants who had COVID-19 to what extent they experienced a list of 29 symptoms during the first two weeks of their infection on a 5-point scale (1 = not at all, 5 = all the time, unvalidated). We then combined the number and severity of COVID-19 symptoms, and type of medical care used because of COVID-19 (none, GP, hospital admission, or intensive care admission, unvalidated) into one Z-score.

We quantified COVID-19-related worrying by asking to what extent people were worried about their own health during the first two weeks of their infection on a 5-point scale (1 = never, 5 = all the time, unvalidated). Use of COVID-19-related aftercare was defined by whether the participants received physical therapy, ergotherapy, social care or were referred to a neurologist, pulmonologist, psychologist, or psychiatrist after their COVID-19 infection (yes, or no, unvalidated).

Finally, we measured pandemic-related stressor load with a questionnaire asking whether and to what extent participants experienced a list of 18 potential stressors on a 6-point scale (1 = not bothersome, 6 = very bothersome) (van der Heide et al., n.d.).

### 2.7 Statistical analysis

#### 2.7.1 Data quality checks

As preregistered, we excluded participants who accepted more than 90 % or less than 10 % of the offers in the decision phase, as this indicated that participants did not understand or execute the task as expected. To make sure this criterium did not introduce bias by excluding the least or most fatigued participants, we tested whether overall acceptance rate was related to fatigue using Pearson's correlation test. In addition, we excluded participants who pressed the same button in > 70 % of the decision trials, who missed more than 10 decision trials, whose MBT was < 10, or whose mean reaction time was shorter than 500ms, as this also indicates that participants did not execute the task as expected.

Furthermore, we checked all continuous variables for outliers using visual inspection of histograms and boxplots.

In addition, we calculated the mean scores of items of the NASA task load questionnaire for each group, and tested differences between effort levels and groups using two-way ANOVAs with Tukey-HSD post hoc test.

#### 2.7.2 Demographics

We report demographics (age, sex, educational level, socio-economic status, ethnicity), participant characteristics (smoking, alcohol use, exerce, BMI, chronic disease, current physical health (SF36), fatigue in the past two weeks (MFI)), and COVID-19-related characteristics (symptom severity, time since onset, medical care used, vaccination status, recovery status, worrying during illness) as mean ± SD or proportions. Differences between the three groups were tested with one-way ANOVAs and Tukey-HSD post-hoc tests for continuous variables, or chi-square tests for categorical variables.

#### 2.7.3 Difference in fatigue and depressive mood between groups

We tested whether fatigue and depressive mood scores differed between groups by performing two one-way ANOVAs followed by Tukey-HSD post hoc tests.

#### 2.7.4 Difference in reward and effort sensitivity between groups

We tested whether effort and reward sensitivity differed between groups by using mixed binomial regression analysis with the decision to accept the trial's offer (no/yes) as dependent variable, Reward, Effort and Reward∗Effort as random factors, the interaction between Group (ref: noCOVID), Reward and Effort as fixed factors of interest, and Age and Sex (ref: male) as fixed factors of no interest (model 1a). This model was repeated with a recoded Group variable (ref: >12wkCOVID), to test the differences between all three groups (model 1b). In case of positive findings on model 1, we additionally compared the groups while only including non-recovered participants, to confirm that the findings represent altered effort/reward sensitivity in participants with symptoms.

#### 2.7.5 Reward and effort sensitivity and fatigue/depressive mood

Following the first objective in our preregistration, we studied the relationship between fatigue and depressive mood with reward and effort sensitivity using mixed binomial regression modelling.

To test whether the associations of fatigue with reward and effort sensitivity differed between the groups, we constructed a mixed model with the decision to accept the trial's offer or not (no/yes) as dependent variable, Reward, Effort and Reward∗Effort as random factors, the interaction between Group (ref: noCOVID), Fatigue, Reward and Effort as fixed factors of interest, and Age and Sex (ref: male) as fixed factors of no interest (model 2a). This model was repeated with a recoded Group variable (ref: >12wkCOVID), to test the differences between all three groups (model 2b). Second, we tested whether the associations of depressive mood with reward and effort differed between the groups by replacing Fatigue with Depressive mood scores in the above-described models (model 3a and 3b). We Z-scored all fixed and random variables in the models. For each model, estimates (betas) of Reward and Effort for each participant were derived for interpretation and visualization. These beta's represent reward and effort sensitivity, i.e. the extent to which decisions to accept an offer are affected by reward and effort levels, respectively. For similar interpretation of the betas for Reward and Effort level, we reversed the Effort levels (1 = 90 % MVC, 5 = 10 % MVC) in these regression models such that higher values represent lower effort levels. Now for both Reward and Effort, more positive betas represent higher sensitivity.

#### 2.7.6 Prediction analysis

For our second preregistered objective, we tested the following nine predictors of reward and effort sensitivity: age (years), sex (male, female), pre-pandemic BMI (kg/m2), pre-pandemic lifestyle, socio-economic status, pre-pandemic physical health, COVID-19 disease severity, worrying during the first two weeks of COVID-19, and use of COVID-19-related aftercare. We checked all continuous predictor variables for outliers using visual inspection of histograms and boxplots. For the prediction analysis, we transformed all predictors to Z-scores. First, we tested whether these predictors were related to reward and effort sensitivity within each group by performing three mixed binomial regression models, one for each group, with the trial answers (no/yes) as dependent variable, Reward, Effort and Reward∗Effort as random factors, and the interaction between the nine predictors, Reward level, and Effort level as fixed factors (model 4a, 4b and 4c). We considered the nine variables significant predictors of effort or reward sensitivity if there was a significant interaction with the effort or reward effect on acceptance rates.

Second, to test whether the associations of the predictors with reward and effort sensitivity differed between the three groups, we added Group ref: noCOVID) as fixed factor to this model (model 5a). This model was repeated with a recoded Group variable (ref: >12wkCOVID), to test the differences between all three groups (model 5b). Predictors that were only measured in one or two of the three groups were not included in these models (i.e. COVID-19 disease severity, worrying during the first two weeks of COVID-19, and use of COVID-19-related aftercare). For similar interpretation of the betas for Reward and Effort level, we again reversed the levels of Effort levels (1 = 90 % MVC, 5 = 10 % MVC) in these regression models such that higher values represent lower effort levels.

We considered p < 0.05 significant. We performed regression analyses using RStudio (version 1.4, R Core Team (2021)), R Foundation for Statistical Computing, Vienna, Austria) using the packages lme4 and lmerTest (Bates et al., 2015; Kuznetsova et al., 2017). The R code of all models can be found in Table S1 of Supplementary Material 1.

## 3 Results

### 3.1 Population characteristics

In total, 233 out of the 308 participants who completed the online study were included in the study after pre-registered data quality control (https://osf.io/uhwkc), of which 90 never had COVID-19, 62 had COVID-19 < 4 weeks ago and 81 had COVID-19 > 12 weeks ago. In total, 67 of the 308 participants were excluded because they accepted more than 90 % or less than 10 % of the offers in the decision phase. Fatigue was not related to overall acceptance rate (r = 0.03, p = 0.542). Furthermore, 8 participants were excluded because they pressed only one button in over 70 % of the decision trials (n = 3), they missed more than 10 decision trials (n = 1), their MBT was more than 10 (n = 3), or their mean reaction time was shorter than 500ms (n = 1).

Table 1 and Table S2 of Supplementary Material 1 show the demographic characteristics and reported symptoms of the study population. The COVID-19 > 12 weeks group reported significantly more fatigue (as measured with the MFI) and more limitations in daily functioning (as measured with the SF-36) compared to the COVID-19 < 4 weeks and no COVID-19 groups. However, no group differences were observed in emotional well-being or limitations due to emotional problems. Similarly, the COVID-19 < 4 weeks and COVID-19 > 12 weeks groups reported overall more physical and fatigue-related symptoms compared to participants who did not have COVID-19, while feeling depressed, having low motivation and (pandemic-related) stress did not differ between the three groups. The groups differed on several other demographic, disease-related, and lifestyle factors, including age, BMI, symptom severity, and worrying (see Table 1, and Table S2, Table S3, Figure S2, and Figure S3 of Supplementary Material 1.

**Table 1:** Demographic, physical health, mental health, and COVID-19-related characteristics of the study population according to group.

		Total (n = 242)	No COVID-19 (n = 90)	COVID-19 < 4 weeks (n = 62)	COVID-19 > 12 weeks (n = 81)	
		Mean ± SD or n (%)	Mean ± SD or n (%)	Mean ± SD or n (%)	Mean ± SD or n (%)	P-value
Age (years)		35 ± 13	28 ± 10	36 ± 13	43 ± 12	<0.001^abc^
Sex (% male)		41 (17.7)	21 (23.3)	7 (11.4)	13 (16.0)	0.154
BMI, pre-pandemic (kg/m^2^)		24.3 ± 4.7	22.3 ± 2.9	23.7 ± 3.6	27.0 ± 5.8	<0.001^bc^
Ethnicity (% Dutch)		219 (94.0)	81 (90.0)	58 (93.5)	80 (98.8)	0.054
Education (ISCED score)		5.1 ± 1.5	5.2 ± 1.6	5.4 ± 1.5	4.8 ± 1.3	0.062
Income (€)		50,182 ± 40,978	38,306 ± 38,762	59,314 ± 44,075	56,389 ± 38,138	0.002^ab^
Smoking, pre-pandemic (% daily)		8 (3.4)	3 (3.3)	2 (3.2)	3 (3.7)	0.465
Alcohol use, pre-pandemic (times per month)		4.3 ± 5.1	3.7 ± 4.6	5.7 ± 6.0	3.8 ± 4.7	0.035^a^
Medium intensive exercising, pre-pandemic (h per week)		3.34 ± 1.72	2.90 ± 1.73	3.19 ± 1.56	3.94 ± 1.67	0.001^bc^
High intensive exercising, pre-pandemic (h per week)		2.34 ± 1.72	2.22 ± 1.67	2.48 ± 1.70	2.38 ± 1.80	0.639
Having chronic disease, pre-pandemic (%)		49 (21)	16 (18)	6 (9.7)	22 (27)	0.028^c^
SF-36 (range 0–100)						
	Physical functioning	73 ± 28	91 ± 13	74 ± 24	51 ± 27	<0.001^abc^
	Role limitations due to physical health	43 ± 45	77 ± 34	33 ± 41	14 ± 32	<0.001^abc^
	Role limitations due to emotional problems	58 ± 44	57 ± 42	59 ± 46	58 ± 44	0.970
	Energy/fatigue	43 ± 23	54 ± 19	42 ± 23	32 ± 20	<0.001
	Emotional well-being	62 ± 20	63 ± 20	63 ± 22	61 ± 17	0.751
	Social functioning	49 ± 26	57 ± 21	48 ± 28	41 ± 29	<0.001^b^
	Pain	71 ± 27	86 ± 19	67 ± 26	57 ± 27	<0.001^abc^
	General health	57 ± 20	65 ± 18	61 ± 20	46 ± 19	<0.001^bc^
MFI						
	Total fatigue (range 0–100)	66.9 ± 19.4	57.4 ± 17.0	67.9 ± 19.8	76.7 ± 16.6	<0.001^abc^
	General fatigue (range 0–20)	14.8 ± 4.4	12.4 ± 4.0	15.1 ± 4.0	17.3 ± 3.7	<0.001^abc^
	Physical fatigue (range 0–20)	13.9 ± 5.1	11.4 ± 4.5	14.0 ± 4.7	16.7 ± 4.5	<0.001^abc^
	Reduced motivation (range 0–20)	11.2 ± 4.0	10.0 ± 3.6	11.7 ± 4.8	12.0 ± 3,6	<0.002^ab^
	Reduced activity (range 0–20)	13.9 ± 4.4	11.9 ± 4.1	14.5 ± 4.4	15.5 ± 4.1	<0.001^ab^
	Mental fatigue (range 0–20)	13.1 ± 4.8	11.6 ± 4.6	12.7 ± 4.6	15.1 ± 4.5	<0.001^bc^
**COVID-19-related information**						
COVID-19-related stressors (score, range 0–125)¥		40 ± 11	38 ± 9	41 ± 12	41 ± 11	0.078
Time since symptom onset (days)		112 ± 129	N.a.	15 ± 27	190 ± 125	<0.001
Symptom severity during first two weeks (score, range 0–89)		45.3 ± 22.4	N.a.	38.6 ± 21.3	50.5 ± 22	0.001
Medical care used						<0.001
	*None*	72 (50.7)	N.a.	47 (75.8)	25 (31.3)	
	*GP/Emergency unit*	63 (44.4)	N.a.	15 (24.2)	48 (60.0)	
	*Hospital admission*	6 (4.2)	N.a.	0 (0.0)	7 (8.7)	
Aftercare (% yes)		66 (46.2)	N.a.	6 (9.7)	60 (74.1)	<0.001
Vaccinated before COVID-19 infection (% yes)		36 (60.0)	N.a.	34 (94.4)	1 (4.3)	<0.001
Not yet recovered at moment of participation (% yes)		105 (73.9)	N.a.	38 (61.3)	67 (83.8)	0.004
Worrying about illness in first two weeks of infection (score, range 1–6)		3.9 ± 1.3	N.a.	3.8 ± 1.4	4.0 ± 1.2	0.419

BMI, body mass index; GP, general practitioner; ISCED, International Standard Classification of Education; MFI, Multidimensional Fatigue Index; SF36, 36-Item Short Form Health Survey; POMS, Profile of Mood States.

P-values for differences between COVID-19 groups were determined by performing a one-way ANOVA with Tukey-HSD post hoc test for continuous variables and a chi-square test for categorical variables.

^a^No COVID-19 group differs significantly from the COVID-19 < 4 weeks group (P < 0.05). ^b^No COVID-19 group differs significantly from the COVID-19 > 12 weeks group (P < 0.05). ^c^COVID-19 < 4 weeks group differs significantly from the COVID-19 > 12 weeks group (P < 0.05). ¥ Scores of all individual stressors can be found in Table S3 of Supplementary Material 1.

### 3.2 Fatigue and depressive mood at time of testing

**Fig. 2:**
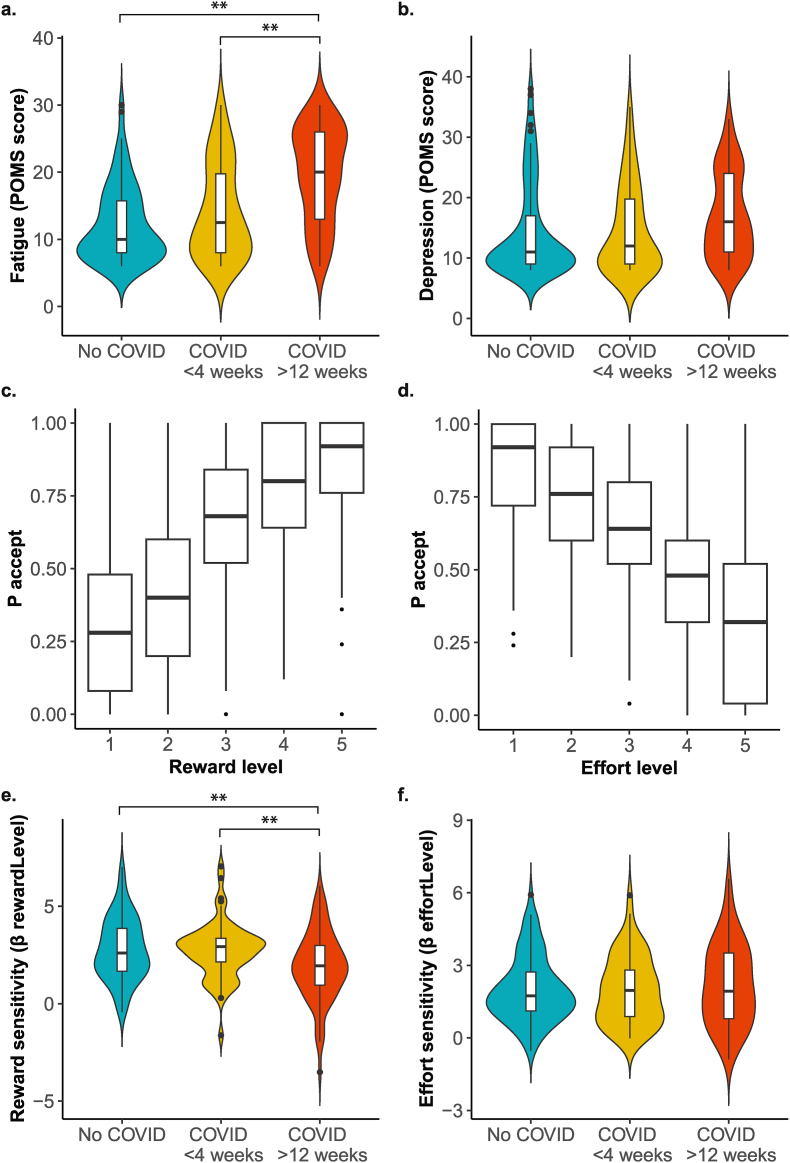
State fatigue (a) and depressive mood (b) scores at moment of participation according to group and mean acceptance rates for each reward (c) and effort (d) level during the decision phase of the effort-based decision-making task, and therefrom derived values for reward sensitivity (e), effort sensitivity (f) according to group. Regression coefficients for reward (e) and effort (f) were determined by mixed model binomial regression analysis. ∗∗P < 0.01.

The COVID-19 > 12 weeks group was more fatigued at time of testing compared to the COVID-19 < 4 weeks and no COVID-19 groups (mean ± SD: 20 ± 7 vs. 14 ± 7 and 12 ± 6 POMS-score, respectively; both p < 0.001) (Fig. 2a). Depressive mood at time of testing did not differ between the groups (p = 0.052) (Fig. 2b).

### 3.3 Effort-based decision-making task: validation

**Fig. 3:**
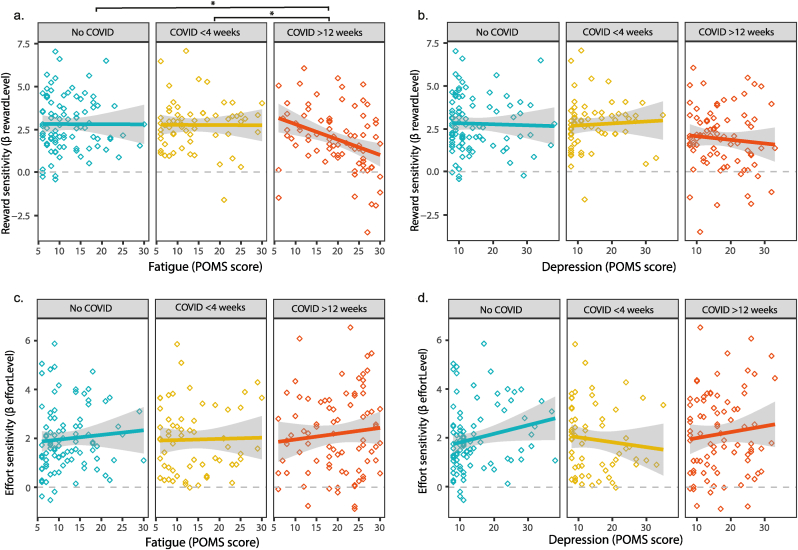
The association between fatigue and depressive mood with reward (a and b) and effort (c and d) sensitivity according to group. The regression coefficients for reward and effort sensitivity, and the differences between groups were determined and tested by mixed model binomial regression analysis. A higher regression coefficient reflects more optimal decision making for reward or effort, i.e. higher reward or effort sensitivity. ∗P < 0.05.

Participants accepted more offers when the reward was high or when the required effort level was low (Fig. 3c and d). The mixed binomial regression analysis showed a main effect of Reward (β 2.66 (95 % confidence interval (95 %CI): 2.40, 2.93); OR: 14.30 (95 %CI: 11.02, 18.73), p < 0.001) and Effort (β 2.21 (95 %CI: 1.98, 2.45); OR: 9.11 (95 %CI: 7.24, 11.59), p < 0.001) across the three groups. This is consistent with previous work using a similar task (Bonnelle et al., 2016). The results of NASA task load Index were in line with this pattern: participants experienced the increasing effort levels as more effortful (F = 95.974, df = 4, p < 0.001), more physically (F = 65.555, df = 4, p < 0.001), mentally (F = 67.988, df = 4, p < 0.001) and time demanding (F = 158.900, df = 4, p < 0.001), and frustrating (F = 51.743, df = 4, p < 0.001), and these ratings did not differ between the groups (Fig. S4, Supplementary Material 1). We also did not observe differences between the groups in the ability to perform the task (Fig. S5, Supplementary Material 1).

### 3.4 Effort-based decision-making task: group differences

Reward sensitivity, the extent to which increasing reward levels affect the choice to accept the offer, was lower in the COVID-19 > 12 weeks group compared to the no COVID-19 group (Fig. 2e; Reward∗Group(COVID>12weeks vs. noCOVID): β −1.05 (95 % CI -1.63, −0.47), OR: 0.35 (95 %CI 0.20, 0.62), p < 0.001) and compared to the COVID-19 < 4 weeks group (Reward∗Group(COVID>12weeks vs. COVID<4weeks): β −0.97 (95 % CI -1.61, −0.34); OR: 0.38 (95 %CI 0.20, 0.72), p = 0.003) (model 1).

Effort sensitivity, i.e. the extent to which increasing effort levels affect the choice to accept the offer, did not differ between the groups (Fig. 2f, model 1).

In the non-recovered population, we found similar group differences for reward sensitivity (Fig. S6, Supplementary Material 1).

### 3.5 Effort-based decision-making task: relationship between fatigue and reward/effort sensitivity

In addressing our first objective, we found that the association between fatigue and reward sensitivity was more negative in the COVID-19 > 12 weeks group compared to the no COVID-19 and the COVID-19 < 4 weeks group, i.e. higher fatigue was more strongly associated with lower reward sensitivity in the COVID-19 > 12 weeks group than in the no COVID-19 and COVID-19 < 4 weeks group (Fig. 3; β Reward∗Fatigue∗Group (COVID>12weeks vs. noCOVID): −0.74 (95 % CI -1.39, −0.08): OR 0.48 (95 %CI 4.01, 0.92), p = 0.029; β Reward∗Fatigue∗Group (COVID>12weeks vs. COVID<4weeks): −0.75 (95 % CI -1.40, 0.12); OR: 0.47 (95 %CI 0.25, 1.13), p = 0.022, respectively) (model 2). Visual inspection of the regression plots suggests that, especially in high-fatigued individuals in the COVID-19 > 12 weeks group, the impact of reward information on decisions is lower compared to the no COVID-19 and the COVID-19 < 4 weeks groups, while those reporting low fatigue showed reward sensitivity levels comparable to the other groups. Fatigue was not associated to reward sensitivity across groups (no Reward∗Fatigue interaction: β −0.01 (95 % CI -0.52,0.49); OR 0.99 (95 %CI 0.60, 1.64), p = 0.958).

No Effort∗Fatigue∗Group interaction was observed, indicating that the relationship between fatigue and effort sensitivity did not differ between groups (all β < 0.39, all OR < 1.47, all p > 0.05) (model 2). We also did not observe interactions between Effort∗Fatigue, indicating that fatigue was not associated with effort sensitivity across the three groups (β Effort∗Fatigue: 0.15 (95 % CI -0.32,0.63): OR 1.16 (95 %CI 0.73, 1.87), p = 0.520). The results of the full binomial mixed regression model can be found in Table S4 of Supplementary Material 1.

### 3.6 Effort-based decision-making task: relationship between depressive mood and reward/effort sensitivity

For the second part of our first objective, we found that the relationship between depressive mood and reward or effort sensitivity did not differ between the groups (all β < 0.49, all OR < 1.63, all p > 0.05 for the Reward∗DepressiveMood∗Group and Effort∗DepressiveMood∗Group interactions) (model 3). We also did not observe a Reward∗DepressiveMood or Effort∗DepressiveMood interaction, indicating that, across the three groups, depressive mood was not associated with reward or effort sensitivity (Reward∗DepressiveMood: −0.04 (95 % CI -0.43, 0.66): OR 0.96 (95 %CI 0.65, 1.42), p = 0.841; β Effort∗DepressiveMood: 0.32 (95 % CI -0.03, 0.66): OR 1.37 (95 %CI 0.97, 1.94), p = 0.075) (model 3). The results of the full binomial mixed regression model can be found in Table S4 of Supplementary Material 1.

### 3.7 Effort-based decision-making task: predictors of reward and effort sensitivity in the COVID-19 > 12 weeks group

Next, for our secondary aim, we identified risk factors of reward and effort sensitivity. Here, we focused on the associations of the predictors with reward or effort sensitivity within the COVID-19 > 12 weeks group and how they differed from the other two groups. In this analysis, one participant in the no COVID and one participant in the COVID > 12 week group were excluded because of suspected erroneous input, which were considered outliers. The assumptions for multiple regression, including the absence of multicollinearity, were met.

**Fig. 4:**
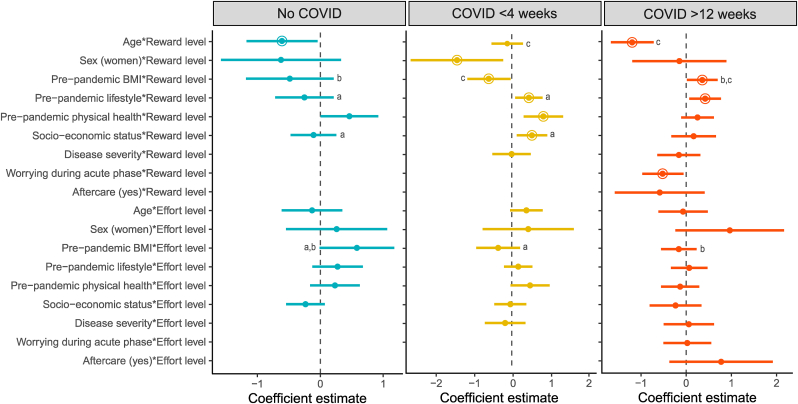
Prediction regression models of reward and effort sensitivity in each group. Regression coefficients were estimated using mixed model binomial regression analysis. Continuous predictors were Z-scored. Error bars represent the 95 % confidence interval. Significant regression coefficients are highlighted by a circle (P < 0.05). A higher regression coefficient for reward or effort reflects more optimal decision making for reward or effort, i.e. higher reward or effort sensitivity. ^a^No COVID-19 group differs significantly from the COVID-19 < 4 weeks group (P < 0.05). ^b^No COVID-19 group differs significantly from the COVID-19 > 12 weeks group (P < 0.05). ^c^COVID-19 < 4 weeks group differs significantly from the COVID-19 > 12 weeks group (P < 0.05).

Regarding reward sensitivity, we found that in the COVID-19 > 12 weeks group, higher age and worrying during the first two weeks of COVID-19 were associated with lower reward sensitivity, while a higher pre-pandemic BMI and a healthier pre-pandemic lifestyle were associated with higher reward sensitivity (β Age∗Reward: −1.20 (95 %CI -1.68, −0.73); OR 0.30 (95 %CI 0.19, 0.48), p < 0.001); β Worrying∗Reward: −0.52 (95 %CI -0.98, −0.07); OR 0.59 (95 %CI 0.38, 0.94), p = 0.025; β BMI∗Reward: 0.35 (95 %CI 0.01, 0.69); OR 1.43 (95 %CI 1.01, 2.00), p = 0.047; β Lifestyle∗Reward: 0.41 (95 %CI 0.06, 0.76); OR 1.50 (95 %CI 1.06, 2.14), p = 0.022)) (Fig. 4, model 4). When comparing the groups, we found that in the COVID-19 > 12 weeks group, age was more negatively associated with reward sensitivity compared to the COVID-19 < 4 weeks group (β Age∗Reward∗Group(COVID-19 > 12 weeks vs. COVID-19 < 4 weeks): 0.86 (95 % CI 0.13, 1.58); OR 2.36 (95 %CI 1.14, 4.88), p = 0.020) (model 5). In addition, in the COVID-19 > 12 weeks group, BMI was more positively associated with reward sensitivity compared to the COVID-19 < 4 weeks group (β Age∗Reward∗Group(COVID-19 > 12 weeks vs. COVID-19 < 4 weeks): −0.94 (95 % CI -1.61, −0.27); OR 0.39 (95 %CI 0.20, 0.76), p = 0.006) (model 5). No other differences between the COVID-19 > 12 weeks group and the other two groups were observed for the relationships between reward sensitivity and any of the other predictors (all p > 0.05).

Regarding effort sensitivity, we did not find significant predictors of effort sensitivity within the COVID-19 > 12 weeks group (Fig. 4, model 4). However, when comparing the groups, we found that, pre-pandemic BMI was more negatively associated with effort sensitivity in the COVID-19 > 12 weeks group, compared to the no COVID-19 group (β BMI∗Effort∗Group(COVID-19 > 12 weeks vs. no COVID): −0.73 (95 %CI -1.45, −0.01); OR 0.48 (95 %CI 0.23, 0.99), p = 0.048)) (model 5).

The correlation matrix of the different predictors across groups can be found in Fig. S7 of Supplementary Material 1. Regression plots of the significant associations between the predictors and effort or reward sensitivity for each group are shown in Fig. S8 of Supplementary Material 1.

## 4 Discussion

People with persisting symptoms after COVID-19 commonly report fatigue and depressive mood as primary symptoms. However, it is unclear how these symptoms relate to decisions regarding effortful and rewarding activities, and whether this differs from fatigue and depression experienced during the early COVID-19 phase. Here, for our first objective, we investigated how self-reported fatigue and depressive mood during task performance were associated with different components of decision making (i.e. reward and effort sensitivity) in three groups: a group who had COVID-19 > 12 weeks ago, a group who had COVID-19 < 4 weeks ago and a group who never had COVID-19. In addition, for our second objective, we aimed to identify which risk factors were associated with reward and effort sensitivity in the > 12 weeks group.

Results indicate that higher self-reported fatigue > 12 weeks after COVID-19 is associated with lower reward, but not effort, sensitivity during decision making, while depressive mood was not associated with either of these decision-making parameters. In addition, higher age, a less healthy lifestyle, a lower pre-pandemic BMI, and more worrying during the first two weeks of COVID-19 were predictors of lower reward sensitivity in the COVID-19 > 12 weeks group. These results provide valuable insights into how persisting fatigue after COVID-19 may impact decision making in daily functioning.

Participants in the > 12 weeks group showed on average lower reward sensitivity during decision making than the < 4 weeks or no COVID-19 groups and this reduction in reward sensitivity was most prominent when fatigue was high. This finding suggests that persisting fatigue > 12 weeks after COVID-19 may involve problems in reward processing, potentially due to downstream effects of the infection on specific neural processes. In line with this, several lines of research suggest that post-COVID-19 may involve persistent (neuro) inflammation, indicated by increased levels of pro-inflammatory cytokines such as TNF-α, IL-1β, and IL-17A (Ceban et al., 2022; Monje and Iwasaki, 2022). These cytokines can cross the blood-brain barrier or signal via vagal pathways to activate microglia and astrocytes, leading to functional changes in cortical and subcortical brain networks that are involved in motivation and reward processing (Guedj et al., 2021; Sollini et al., 2021). More specifically, it has been shown that chronic, but not acute, inflammation can induce long-lasting changes in neural reward processing (Capuron et al., 2012; Felger and Lotrich, 2013). Chronic inflammation has been linked to a reduction in the availability of dopamine receptors and impaired release of dopamine via direct effects of cytokines on the co-factor BH4, needed for the formation of L-DOPA from phenylalanine and tyrosine, a precursor of dopamine (Felger, 2017), thereby diminishing reward sensitivity. In line with this, a study from Lamontagne et al. (2021) observed anhedonia or a loss of interest in previously rewarding activities in post-COVID-19 patients compared to healthy controls. More recently, Zhu et al. (2025) provided evidence for a pathway in which inflammation (IL-6) decreases mesolimbic dopamine in a cancer mouse model, and that apathy and depressive symptoms in the mice could be alleviated by blocking the IL-6 receptors in the brain (Zhu et al., 2025). Accordingly, these studies suggest that the observed lower reward sensitivity in high-fatigued individuals > 12 weeks after COVID-19 may reflect alterations in neural reward processing resulting from long-term effects of (neuro)inflammation. This may have implications for understanding motivational changes in post-COVID-19 patients, as they may result in long-term immune-induced biological changes within the brain. To further confirm this suggestion, future neuroimaging studies could investigate how altered decision making in post-COVID-19 condition is related to neuro-inflammatory processes and dopamine network function.

Furthermore, the finding that persisting fatigue is related to lower reward sensitivity offers insight into how fatigue affects decisions to engage in physical or cognitive activities. A recent review concluded that while self-reported fatigue was not reliably related to performance decline, it was much better captured by the participants’ choices to engage in effortful tasks for rewards and a model that included changes in metabolic states (Pessiglione et al., 2025). Our finding may implicate that these choices are not only affected by the energy costs of those activities, but also by a reduced sensitivity to the potential enjoyment it brings. Identifying these changes in either effort or reward sensitivity could help to better characterize fatigue symptoms of patients, and potentially to identify treatment targets.

Reward sensitivity was, in contrast to our hypothesis, not associated with depressive mood, which also involves anhedonia and loss of interest in rewarding activities. Nor did the relationship between depressive mood and reward sensitivity differ between the groups. This might be due to the fact that depressive mood did not differ between the three groups, both during task performance and in the two weeks prior to participation. Additionally, all groups reported a similar increase in depressive symptoms compared to before the COVID-19 pandemic. Accordingly, this increase in depressive symptoms across all groups might suggest that depressive symptoms may not result from the COVID-19 infection, but may instead reflect a general response to pandemic-related societal changes, such as lockdowns and the overall stress associated with the global health crisis (COVID-19 Mental Disorders Collaborators, 2021; Wu et al., 2021). Previous studies of the symptom profile of COVID-19 also reported that while depression is a prevalent characteristic of post-COVID-19 in 12 % of the cases, fatigue was more prevalent, occurring in 58 % of the cases (Lopez-Leon et al., 2021). Therefore, it is possible that fatigue in the > 12 weeks group is a more direct consequence of the infection and may more readily reflect infection-related effects on neural reward processing, while depressive mood may reflect the general emotional response to the pandemic-related restrictions that may not involve infection-related effects on neural reward processing.

The relationship between fatigue and effort sensitivity did not differ between the groups, in contrast to our hypothesis. We expected the results in the < 4 weeks and > 12 weeks group to be similar to those seen after well-controlled acute immune manipulations using LPS (Draper et al., 2018; Lambregts et al., 2023), which show that fatigue during 1–3 h after LPS administration is associated with increased effort sensitivity. It is possible that this relationship with effort sensitivity only surfaces during the first acute response to illness and that the level of sickness in the < 4 weeks group was too variable. Some participants might have already recovered within that time, while others might not even have signed up for participation because they felt too ill. Given that this was an online experiment, we were not able to control this. Future studies could assess decision making sooner after initial infection and assess inflammatory markers such as C-reactive protein to make sure that the task is performed during the acute phase of sickness.

Alternatively, it might be possible that fatigue is related to effort sensitivity irrespective of disease status. Previous studies have shown that in healthy individuals, momentary fluctuations in state fatigue during task performance are linked to increased effort sensitivity during subsequent decisions to either engage in the task or rest (Matthews et al., 2023; Müller and Apps, 2019). In our study, however, we did not observe a significant association between fatigue and effort sensitivity across the groups. These groups included healthy people, people who were acutely ill, people who were recovered from COVID-19, and people with post-COVID syndrome. This population variation may have obscured potential effects of state fatigue on effort sensitivity. Additionally, unlike the prior studies, our design separated the decision phase from the task execution phase to minimize fatigue effects due to task engagement, which could reduce comparability with findings in healthy individuals. Nevertheless, effort sensitivity did not differ between the groups. However, the findings with respect to reward sensitivity still indicate that the fatigue experienced in the > 12 weeks phase may be driven by different biological mechanisms as compared to the early phase, possibly involving altered dopamine transmission due to an extended period of raised inflammation levels (Guedj et al., 2021; Sollini et al., 2021).

We identified several risk and protective factors of reward sensitivity in this study. Higher age was associated with decreased reward sensitivity in the COVID-19 > 12 weeks and the no COVID-19 groups. This is in line with previous neurocognitive studies, in which age was associated with lower reward sensitivity irrespective of infection status (Dhingra et al., 2020; Eppinger et al., 2012; Kardos et al., 2017). With age, the brain becomes more vulnerable to the inflammatory and neurological effects of COVID-19 (Keller et al., 2022) and may respond with more neuroinflammation following peripheral infection (Tizenberg et al., 2021). This might explain the stronger link between age and reward sensitivity in the COVID-19 > 12 weeks group compared to the other groups.

Furthermore, we found that a healthier lifestyle was protective against lower reward sensitivity in people who had COVID-19 > 12 weeks, which is in line with studies showing that unhealthy lifestyle factors, e.g. low exercise and smoking, are related to higher disease severity of acute COVID-19 and high risk of post-COVID-19 (Bai et al., 2022; Kofahi et al., 2022; Patanavanich and Glantz, 2020; Pływaczewska-Jakubowska et al., 2022; Subramanian et al., 2022). Lifestyle factors like smoking and low exercise have been associated with low-grade inflammation (Teixeira de Lemos et al., 2011; Walsh et al., 2011; Yanbaeva et al., 2007), possibly contributing to the inflammatory effects of (post) COVID-19 on the brain's reward networks.

Notably, while a higher BMI was related to increased sensitivity to rewards in the COVID-19 > 12 weeks group, it was associated with decreased sensitivity to rewards in the COVID-19 < 4 weeks group. This suggests that the mechanisms through which BMI and decision making interact differs between early and long-term phases of COVID-19. The result in the COVID-19 > 12 weeks group parallels that seen in healthy populations where higher BMI is linked to increased sensitivity to rewards (Miras et al., 2012; Sutton et al., 2022). Specifically, high reward sensitivity in obesity has been suggested to contribute to the engagement in eating behaviours. By contrast, the link between BMI and decreased reward sensitivity in the < 4 weeks group is consistent with findings that BMI is positively linked to higher inflammation and higher symptom severity during acute COVID-19 (Kofahi et al., 2022; Subramanian et al., 2022; Vassilopoulou et al., 2022; Zhou et al., 2021), while for post-COVID-19 this link is less clear (Bai et al., 2022; Townsend et al., 2020). We therefore hypothesize that stronger reductions in reward sensitivity in individuals with high BMI during the early phase of COVID-19 might be explained by more severe sickness symptoms and inflammation compared with those with low BMI. Future studies are needed to confirm this hypothesis about the role of BMI in COVID-19.

Finally, we found that worrying during the first two weeks of COVID-19 was associated with less sensitivity to rewards, which is in line with the observation that anxiety contributes to post-COVID-19 fatigue (Calabria et al., 2022). Worrying could reflect increased stress levels and anxiety, potentially related to higher symptom severity, uncertainty of the disease course, and/or limited access to medical care during the acute infection, as well as psychological stressors of the pandemic itself. Such acute stressors can upregulate inflammatory markers and together with an acute infection dysregulate immune function and trigger neuroinflammation (Slavich and Irwin, 2014). On the other hand, as the causal relationship in this study could not be established, participants with lower reward sensitivity already before the infection could also be more prone to develop persistent symptoms. These stress-immune interactions have been proposed to underlie development of depression and its causal relationship should therefore be further investigated in future research.

Together, these findings show that older age, an unhealthy lifestyle, and more worrying during the first phase of COVID-19 may increase the risk of altered reward sensitivity, which underscores the importance of a multifaceted approach in the understanding of and the treatment of post-COVID-19-related symptoms.

This study had several limitations. First, the population in the > 12 weeks group was a mix of recovered individuals and individuals with a diagnosis of post-COVID-19, but we do not know who had a diagnosis for post-COVID-19. We can therefore not conclude that the reduction in reward sensitivity is a characteristic of post-COVID syndrome or merely fatigue after COVID-19. However, there are several factors indicating that at least part of our population was dealing with (post) COVID-19 symptomatology. Participants in the COVID-19 > 12 weeks group were recruited from social media support groups for post-COVID-19 with the goal to bias recruitment to include more participants with post-COVID-19-related fatigue. As expected with this recruitment strategy, participants in the COVID-19 > 12 weeks group were not only more fatigued at the moment of participation, they also reported to be more fatigued in the two weeks prior to participation compared to the other two groups, as measured by the MFI. In addition, more than 80 % of the participants who had COVID-19 > 12 weeks ago reported that they were not yet recovered from their COVID-19 infection, thereby meeting the WHO-definition of post-COVID-19 (Soriano et al., 2022). It is therefore likely that our results relate to the proposed neurobiological mechanisms of post-COVID-19 syndrome (Peluso and Deeks, 2024). To further confirm this, future studies could assess decision making in a properly diagnosed population and compare it to other chronic diseases that involve fatigue and healthy individuals.

Second, our recruitment method also resulted in a selection bias, as seen in demographical differences across groups. Participants in the COVID-19 > 12 weeks group were older, had a higher BMI, had more chronic diseases, and experienced more severe symptoms during the first two weeks of COVID-19. Additionally, the COVID-19 < 4 weeks group and the COVID-19 > 12 weeks group differed in vaccination status, due to the period in which infection of the participant took place. While participants in the > 12 weeks group had COVID-19 mostly before the national vaccination campaign, participants in the < 4 weeks group had COVID-19 after the start of the campaign. These group differences could have created more variability in the > 12 weeks group and these results should therefore be interpreted with caution. Future studies should carefully control and match the different groups on these demographic characteristics.

Third, several variables include in the predictor analysis, e.g. BMI, physical exercise, smoking behaviour, alcohol use before the pandemic, and disease severity and worrying during the early phase of their infection, relied on the participants’ retrospective self-reports of a period ranging from several weeks up to two years. This could have introduced recall bias. For lifestyle-related factors, the reference period was consistent across groups, as all were asked to recall their lifestyle prior to March 2020. However, the timing of disease-related recollections varied between groups: participants in the COVID > 12 weeks group reported on infections that occurred earlier than those in the COVID < 4 weeks group. This should be taken into account when interpreting the predictor results. Future studies should use longitudinal designs to minimize these recall biases.

Fourth, one of the characteristics of the effort-based decision making task used in this study is that is focused on material rewards, while this type of reward may not fully capture the range of motivational processes relevant to decision making in the context of sickness behavior. For instance, in line with our findings regarding reward sensitivity, studies on social reward show that inflammation leads to more social avoidance, an adaptive response to limit infection transmission and to promote recovery (Eisenberger et al., 2017). This highlights the need for future studies to incorporate other types of rewards, such as social rewards, which might help to understand the ecological validity of these findings.

Finally, we used a measure of state fatigue (i.e. fatigue at the moment of testing) rather than trait fatigue (i.e. fatigue in the past two weeks) in our analysis. As in previous studies, we used state fatigue as this relates better to current task performance compared to trait fatigue (van der Schaaf et al., 2018). Moreover, not all participants in the COVID-19 < 4 weeks group had COVID-19 for more than 2 weeks, meaning that the trait fatigue score (which measured across the past two weeks) could not be used for them.

In conclusion, we found evidence that fatigue > 12 weeks after COVID-19 infection, but not depressive mood, is more strongly associated with lower reward sensitivity than in the early phase of COVID-19 (< 4 weeks after COVID). In addition, higher age, unhealthy lifestyle, and worrying during the early phase of COVID-19 are potential risk factors for developing lower reward sensitivity after > 12 weeks. These results indicate that assessment of decision making can provide valuable insights into the characteristics of COVID-19-related fatigue which may ultimately lead to new treatment targets (Pessiglione et al., 2025). Given our finding that symptoms of fatigue > 12 weeks after COVID-19 were associated with reduced reward sensitivity, treatments targeting the neural reward system might provide valuable alternatives or additions to current treatment strategies. One avenue might be pharmacological stimulation of dopamine transmission with agonists or reuptake inhibitors or its downstream inflammatory pathways (Zhu et al., 2025). To progress towards such treatments, future studies should further investigate potential neuroimmune processes that affect dopaminergic reward processing after COVID-19 infections.

## CRediT authorship contribution statement

**Judith M. Scholing:** Writing – original draft, Project administration, Methodology, Formal analysis. **Britt I.H.M. Lambregts:** Writing – review & editing, Methodology. **Ruben van den Bosch:** Writing – review & editing, Supervision, Formal analysis. **Esther Aarts:** Writing – original draft, Supervision, Methodology, Funding acquisition, Conceptualization. **Marieke E. van der Schaaf:** Writing – original draft, Supervision, Methodology, Conceptualization.

## Funding

This project has received funding from the European Research Council (ERC) under the European Union's Horizon 2020 research and innovation programme (grant agreement No 852189) to Esther Aarts.

## Declaration of competing interest

The authors declare the following financial interests/personal relationships which may be considered as potential competing interests: Esther Aarts reports financial support was provided by the 10.13039/501100000781European Research Council. If there are other authors, they declare that they have no known competing financial interests or personal relationships that could have appeared to influence the work reported in this paper.

## Data Availability

Data will be made available on request.
