# Supplementary material for: Greater fatigue is more strongly associated with reduced reward sensitivity in the long-term phase of coronavirus disease (COVID-19) than in the early phase
Source: Brain Behav Immun Health. 2025 Jul 5;48:101056. doi: 10.1016/j.bbih.2025.101056 (PMC12275946; doi:10.1016/j.bbih.2025.101056)
Supplement: Multimedia component 1 [file mmc1.pdf]

Supplementary material 1: Tables and Figures

Title: Greater fatigue is more strongly associated with reduced reward sensitivity in the long-term phase of coronavirus disease (COVID-19) than in the early phase

Authors: Judith M. Scholing[1], Britt I.H.M. Lambregts[1,2], Ruben van den Bosch[1,2], Esther Aarts[1#], Marieke E. van der Schaaf[1,2,3#]

[1]Centre for Cognitive Neuroimaging, Donders Institute for Brain, Cognition and Behaviour, Radboud University, Nijmegen, The Netherlands.

[2]Department of Psychiatry, Radboud University Medical Center, Nijmegen, The Netherlands

[3]Tilburg University, Department of Cognitive Neuropsychology, Tilburg, The Netherlands

[#]These authors contributed equally

Corresponding author: Judith M. Scholing, Judith.scholing@donders.ru.nl

Contents

Supplementary Table S1: R-codes 3

Supplementary Table S2: Pre-pandemic and current health and lifestyle characteristics of the study population. 7

Supplementary Table S3: COVID-related stressor load. 8

Supplementary Table S4: Binomial mixed regression models 10

Supplementary Figure S1: Overview of inclusions of participants who had COVID-19 <4 weeks and >12 weeks ago, and active COVID-19 variants at that moment in the Netherlands 14

Supplementary Figure S2: Physical and psychological symptoms at the moment of participation compared to before the pandemic. 15

Supplementary Figure S3: Physical and psychological symptoms in the first 2 weeks of the acute infection compared to before the pandemic. 16

Supplementary Figure S4: NASA task load according to effort level of the effort-based decision making task 17

Supplementary Figure S5: Maximum number of ticked boxes, success rate of the execution trials and earned apples during the execution phase of the effort-based decision making task according to group 18

Supplementary Figure S6: Fatigue, effort and reward sensitivity in a non-recovered subgroup 19

Supplementary Figure S7: Correlation matrix (Pearson correlations) of predictor and outcome variables across all three groups. 20

Supplementary Figure S8: Scatterplots of the associations of significant predictors with effort and/or reward sensitivity according to group. 21

Supplementary Table S1: R-codes

Model 1: To test whether effort and reward sensitivity differed between groups

Model 1a: Group reference: noCOVID

```
glmer(answer_dich ~
Effort*Reward*Group(ref:noCOVID) + Age + Sex + (1 +
Effort*Reward | subject),
                family = binomial,
                data = data,
                control=glmerControl(optimizer =
c("Nelder_Mead","bobyqa"), optCtrl=list(maxfun=1e+9)))
```

Model 1b: Group reference: <4wkCOVID

```
glmer(answer_dich ~
Effort*Reward*Group_recoded(ref:<4wkCOVID) + Age + Sex + (1 +
Effort*Reward | subject),
                family = binomial,
                data = data,
                control=glmerControl(optimizer =
c("Nelder_Mead","bobyqa"), optCtrl=list(maxfun=1e+9)))
```

Model 2: To test whether the relationship between fatigue and reward and effort on differed between the groups

Model 2a: Group reference: noCOVID

```
glmer(answer_dich ~
Effort*Reward*Fatigue*Group(ref:noCOVID) + Age + Sex + (1 +
Effort*Reward | subject),
                family = binomial,
                data = data_long,
                control=glmerControl(optimizer =
c("Nelder_Mead","bobyqa"), optCtrl=list(maxfun=1e+9)))
```

Model 2b: Group reference: <4wkCOVID

```
glmer(answer_dich ~
Effort*Reward*Fatigue*Group_recoded(ref:<4wkwkCOVID) + Age +
Sex + (1 + Effort*Reward | subject),
                family = binomial,
                data = data_long,
                control=glmerControl(optimizer =
c("Nelder_Mead","bobyqa"), optCtrl=list(maxfun=1e+9)))
```

Model 3: To test whether the relationship between depressive mood and reward and effort differed between the groups

Model 3a: Group reference: noCOVID
```
glmer(answer_dich ~
Effort*Reward*Depression*Group(ref:noCOVID) + Age + Sex + (1 +
Effort*Reward | subject),
                family = binomial,
                data = data_long,
                control=glmerControl(optimizer =
c("Nelder_Mead","bobyqa"), optCtrl=list(maxfun=1e+9)))
```

Model 3b: Group reference: <4wkCOVID
```
glmer(answer_dich ~
Effort*Reward*Depression* Group_recoded(ref:<4wkCOVID) + Age +
Sex + (1 + Effort*Reward | subject),
                family = binomial,
                data = data_long,
                control=glmerControl(optimizer =
c("Nelder_Mead","bobyqa"), optCtrl=list(maxfun=1e+9)))
```

Model 4: To test whether the predictors were related to reward and effort sensitivity within each group

Model 4a: Predictors (subset: noCOVID group)
```
glmer(answer ~
Effort*Reward*Sex +
Effort*Reward*Age +
Effort*Reward*BMI +
Effort*Reward*Lifestyle +
Effort*Reward*PhysicalHealth +
Effort*Reward*socioEconomicStatus +
Effort*Reward*DiseaseSeverity +
Effort*Reward*Aftercare +
Effort*Reward*Worrying +
               (1 + Effort*Reward | subject),
                family = binomial,
                data = data_long[data_long$Group==”noCOVID”,],
                control=glmerControl(optimizer =
c("Nelder_Mead","bobyqa"), optCtrl=list(maxfun=1e+9)))
```

Model 4b: Predictors (subset: <4wkCOVID group)
```
glmer(answer ~
Effort*Reward*Sex +
Effort*Reward*Age +
Effort*Reward*BMI +
Effort*Reward*Lifestyle +
Effort*Reward*PhysicalHealth +
Effort*Reward*socioEconomicStatus +
```

```
Effort*Reward*DiseaseSeverity +
Effort*Reward*Aftercare +
Effort*Reward*Worrying +
               (1 + Effort*Reward | subject),
               family = binomial,
               data =
data_long[data_long$Group==”<4wkCOVID”,],
                control=glmerControl(optimizer =
c("Nelder_Mead","bobyqa"), optCtrl=list(maxfun=1e+9)))
```

Model 4c: Predictors (subset: >12wkCOVID group)

```
glmer(answer ~
Effort*Reward*Sex +
Effort*Reward*Age +
Effort*Reward*BMI +
Effort*Reward*Lifestyle +
Effort*Reward*PhysicalHealth +
Effort*Reward*socioEconomicStatus +
Effort*Reward*DiseaseSeverity +
Effort*Reward*Aftercare +
Effort*Reward*Worrying +
           (1 + Effort*Reward | subject),
             family = binomial,
             data = data_long[data_long$Group==”>12wkCOVID”,],
                control=glmerControl(optimizer =
c("Nelder_Mead","bobyqa"), optCtrl=list(maxfun=1e+9)))
```

<u>Model 5: To test whether the relationship between the predictors and reward and effort sensitivity differed between the three groups</u>

Model 5a: Group reference: noCOVID

```
glmer(answer ~
Effort*Reward*Group(ref:noCOVID)*Sex +
Effort*Reward*Group(ref:noCOVID)*Age +
Effort*Reward*Group(ref:noCOVID)*BMI +
Effort*Reward*Group(ref:noCOVID)*Lifestyle +
Effort*Reward*Group(ref:noCOVID)*PhysicalHealth +
Effort*Reward*Group(ref:noCOVID)*socioEconomicStatus +
Effort*Reward*Group(ref:noCOVID)*DiseaseSeverity +
Effort*Reward*Group(ref:noCOVID)*Aftercare +
Effort*Reward*Group(ref:noCOVID)*Worrying +
               (1 + Effort*Reward | subject),
                 family = binomial,
                 data = data_long,
                 control=glmerControl(optimizer =
c("Nelder_Mead","bobyqa"), optCtrl=list(maxfun=1e+9)))
```

Model 5b: Group reference: <4wkCOVID

```
glmer(answer ~
Effort*Reward*Group_recoded(ref:<4wkCOVID)*Sex +
Effort*Reward*Group(ref:<4wkCOVID )*Age +
Effort*Reward*Group_recoded(ref:<4wkCOVID)*BMI +
Effort*Reward*Group_recoded(ref:<4wkCOVID)*Lifestyle +
Effort*Reward*Group_recoded(ref:<4wkCOVID)*PhysicalHealth +
Effort*Reward*Group_recoded(ref:<4wkCOVID)*socioEconomicStatus
+
Effort*Reward*Group_recoded(ref:<4wkCOVID)*DiseaseSeverity +
Effort*Reward*Group_recoded(ref:<4wkCOVID)*Aftercare +
Effort*Reward*Group_recoded(ref:<4wkCOVID)*Worrying +
               (1 + Effort*Reward | subject),
                 family = binomial,
                 data = data_long,
                 control=glmerControl(optimizer =
c("Nelder_Mead","bobyqa"), optCtrl=list(maxfun=1e+9)))
```

Supplementary Table S2: Pre-pandemic and current health and lifestyle characteristics of the study population according to group.

	Pre-pandemic				Current				Change			
	No COVID-19 (n=90)	COVID-19 <4 weeks (n=62)	COVID-19 >12 weeks (n=81)		No COVID-19 (n=90)	COVID-19 <4 weeks (n=62)	COVID-19 >12 weeks (n=81)		No COVID-19 (n=90)	COVID-19 <4 weeks (n=62)	COVID-19 >12 weeks (n=81)	
	Mean± SD or n (%)	*Mean± SD or n (%)*	*Mean± SD or n (%)*	*P-value*	*Mean± SD or n (%)*	*Mean± SD or n (%)*	*Mean± SD or n (%)*	*P-value*	*Mean± SD or n (%)*	*Mean± SD or n (%)*	*Mean± SD or n (%)*	*P-value*
BMI (kg/m2)	24.3±4.7	22.3±2.9	27.0±5.8	<0.001[bc]	24.6±4.9	22.6±3.0	27.5±5.9	<0.001[bc]	0.35±1.03	-0.02±1.58	0.77±2.19	0.019[c]
Smoking (% daily)	3 (3.3)	2 (3.2)	3 (3.7)	0.465	2 (2.2)	2 (3.2)	1 (1.2)	0.938	-1 (-1.1)	0 (0.0)	-2 (-2.5)	n.a.
Alcohol use (times per month)	3.7±4.6	5.7±6.0	3.8±4.7	0.035[a]	3.2±4.4	3.4±4.0	2.1±3.7	0.111	-0.5±3.8	-2.3±5.4	-1.7±3.3	0.022[a]
Medium intensive exercising (h per week)	2.90±1.73	3.19±1.56	3.94±1.67	0.001[bc]	2.70±1.65	1.39±1.39	2.31±1.64	<0.001[ac]	-0.20±2.08	-1.80±1.76	-1.63±2.01	<0.001[ab]
High intensive exercising (h per week)	2.22±1.67	2.48±1.70	2.38±1.80	0.639	1.46±1.48	0.55±1.11	1.10±1.47	<0.001[ac]	-0.77±1.65	-1.94±1.76	-1.28±2.18	0.001[a]
Having chronic disease (%)	16 (18)	6 (9.7)	22 (27)	0.028[c]	16 (18)	7 (11)	26 (32)	0.006[bc]	0 (0.0)	1 (1.6)	3 (1.2)	n.a.

BMI, body mass index.

P-values for differences between COVID-19 groups were determined by performing a one-way ANOVA with Tukey-HSD post hoc test for continuous variables and a chi-square test for categorical variables.

[a]No COVID-19 group differs significantly from the COVID-19 < 4 weeks group (P < 0.05). [b]No COVID-19 group differs significantly from the COVID-19 > 12 weeks group (P < 0.05). [c]COVID <4 weeks group differs significantly from the COVID-19 > 12 weeks group (P < 0.05).

Supplementary Table S3: COVID-related stressor load according to group.

Stressors	No COVID-19 (n=90)		COVID-19 <4 weeks (n=62)		COVID-19 >12 weeks (n=81)		P-value frequency	P-value burden
	Frequency	Average burden (1-5)	Frequency (%)	Average burden (1-5)	Frequency (%)	Average burden (1-5)		
	n (%)	Mean±SD	n (%)	Mean±SD	n (%)	Mean±SD		
Loss of social contact and social events	86 (96)	2.55±0.87	60 (97)	2.74±1.05	76 (94)	2.65±1.07	0.796	0.616
COVID-19 related media coverage	76 (84)	2.83±1.42	57 (92)	3.35±1.38	76 (94)	3.20±1.36	0.104	0.083
Family, friends, or loved ones working in vital professions	53 (59)	2.74±1.75	38 (61)	2.82±1.76	56 (69)	2.73±1.68	0.361	0.954
Feeling restricted to leave your home	52 (58)	2.23±1.42	56 (90)	2.70±1.14	57 (70)	2.76±1.65	<0.001[ac]	0.053
Family, friends, or loved ones being at increased risk for a serious course of the disease in case of a COVID-19 infection	59 (66)	2.21±1.33	46 (74)	2.38±1.33	58 (72)	2.33±1.35	0.481	0.765
Having COVID-19 symptoms, or symptoms that could be related	33 (37)	1.87±1.47	59 (95)	3.00±1.05	69 (85)	2.60±0.93	<0.001[ab]	<0.001[ab]
Being at risk for an infection (e.g., at work, in the supermarket)	54 (60)	2.36±1.53	45 (73)	2.68±1.52	62 (77)	2.63±1.45	0.051	0.414
Not able to perform physical activity as usual	65 (72)	2.37±1.41	54 (87)	2.55±1.18	71 (88)	2.67±1.10	0.014[ab]	0.393
COVID-19 symptoms, or symptoms that could be related, in family members, friends, loved ones or colleagues	50 (56)	2.15±1.48	46 (74)	2.44±1.36	48 (59)	2.20±1.38	0.057	0.478
Conflicts or disagreements in the family, social or professional environment	41 (47)	1.80±1.15	40 (65)	1.93±1.09	49 (61)	2.00±1.21	0.053	0.560
Increased workload or work-related obstacles	36 (40)	1.58±0.92	36 (58)	2.15±1.39	46 (57)	1.81±1.12	0.036[ab]	0.017[a]
Family, friends or loved ones are at the hospital and you are restricted in visiting them	47 (52)	1.82±1.25	28 (45)	1.75±1.22	42 (52)	1.90±1.22	0.649	0.802
Severe disease or psychiatric problems of yourself or a loved one	37 (42)	1.68±1.08	23 (37)	1.49±0.93	43 (54)	1.91±1.29	0.114	0.125
Tensions at home or family conflict	33 (37)	1.63±1.11	32 (52)	2.04±1.39	37 (46)	1.90±1.26	0.173	0.141
Problems with access to healthcare, medication, or sanitation	32 (36)	1.80±1.38	24 (39)	1.73±1.23	42 (52)	2.23±1.56	0.081	0.075
Being at increased risk for a serious course of the disease in case of an infection (belonging to a risk group)	23 (26)	1.60±1.28	24 (39)	1.86±1.46	36 (44)	1.95±1.39	0.030[b]	0.258
Problems obtaining basic needs and services	20 (22)	1.40±0.99	31 (50)	2.12±1.50	30 (37)	1.80±1.33	0.002[ab]	0.003[a]
Financial problems	28 (32)	1.62±1.20	16 (26)	1.52±1.11	22 (28)	1.59±1.25	0.695	0.871
Unable to attend a funeral of a loved one	17 (19)	1.28±0.81	15 (24)	1.45±1.00	22 (27)	1.42±0.85	0.430	0.462
(Threat of) job loss, insolvency of a private company, for yourself or someone in your household	13 (14)	1.13±0.43	8 (13)	1.28±0.88	19 (23)	1.36±0.90	0.172	0.133

Death of a loved one	16 (18)	1.26±0.73	17 (27)	1.27±0.62	10 (13)	1.20±0.63	0.077	0.792
Difficulties combining work with childcare	6 (6.7)	1.10±0.45	9 (15)	1.25±0.77	12 (15)	1.16±0.59	0.176	0.331
Separation of a loved one	7 (8.0)	1.20±0.79	5 (8.1)	1.15±0.68	4 (5.0)	1.13±0.67	0.684	0.774
Total stress load	n.a.	38±9	n.a.	41±12	n.a.	41±11	n.a.	0.078

P-values for differences between COVID-19 groups were determined by performing a one-way ANOVA with Tukey-HSD post hoc test for continuous variables and a chi-square test for categorical variables.

[a]No COVID-19 group differs significantly from the COVID-19 < 4-week group (P < 0.05). [b]No COVID-19 group differs significantly from the COVID-19 > 12-week group (P < 0.05). [c]COVID-19 < 4-weeks group differs significantly from the COVID-19 > 12-week group (P < 0.05).

Supplementary Table S4: Binomial mixed regression models to test whether the relationship between fatigue and reward and effort differed between the groups (objective 1)

Table S5a: To test whether the associations of fatigue with reward and effort differed between the groups (model 2a, ref: noCOVID)

**Characteristic**	**log(OR)**[1]	**95% CI**[1]	**p-value**
**Effort**	2.2	1.8, 2.6	**<0.001**
**Reward**	3.0	2.6, 3.5	**<0.001**
**Group**			
noCOVID	—	—	
<4wkCOVID	-0.31	-1.0, 0.43	0.416
>12wkCOVID	-0.12	-0.91, 0.66	0.757
**Fatigue**	-0.28	-0.82, 0.27	0.318
**Sex**			
Male	—	—	
Female	0.00	-0.65, 0.65	0.997
**Age**	0.04	-0.26, 0.34	0.794
**Effort * Reward**	-0.29	-0.62, 0.05	0.092
**Effort * Group**			
*Effort * <4wkCOVID*	-0.11	-0.72, 0.50	0.714
*Effort * >12wkCOVID*	0.01	-0.60, 0.63	0.970
**Reward * Group**			
*Reward * <4wkCOVID*	-0.08	-0.73, 0.58	0.822
*Reward * >12wkCOVID*	-0.57	-1.2, 0.08	0.087
**Effort * Fatigue**	0.15	-0.32, 0.63	0.520
**Reward * Fatigue**	-0.01	-0.52, 0.49	0.959
**Group * Fatigue**			
*<4wkCOVID * Fatigue*	0.24	-0.53, 1.0	0.544
*>12wkCOVID * Fatigue*	0.23	-0.48, 0.95	0.524
**Effort * Reward * group**			
*Effort * Reward * <4wkCOVID*	0.15	-0.33, 0.64	0.531
*Effort * Reward * >12wkCOVID*	0.08	-0.40, 0.56	0.745
**Effort * Reward * Fatigue**	-0.09	-0.46, 0.29	0.647
**Effort * Group* Fatigue**			
*Effort * <4wkCOVID * Fatigue*	-0.11	-0.76, 0.55	0.751
*Effort * >12wkCOVID * Fatigue*	0.03	-0.58, 0.65	0.913
**Reward * Group * Fatigue**			
*Reward * <4wkCOVID * Fatigue*	0.02	-0.69, 0.72	0.962
*Reward * >12wkCOVID * Fatigue*	-0.74	-1.4, -0.08	**0.029**
**Effort * Reward * Group* Fatigue**			
*Effort * Reward * <4wkCOVID * Fatigue*	0.20	-0.32, 0.72	0.451
*Effort * Reward * >12wkCOVID * Fatigue*	0.39	-0.10, 0.88	0.117

[1]OR = Odds Ratio, CI = Confidence Interval

Table S5b: To test whether the associations of fatigue with reward and effort differed between the groups (model 2b, ref: COVID <4 weeks)

**Characteristic**	**log(OR)**[1]	**95% CI**[1]	**p-value**
**Effort**	2.1	1.7, 2.6	**<0.001**
**Reward**	2.9	2.5, 3.4	**<0.001**
**Group_recoded**			
<4wkCOVID	—	—	
noCOVID	0.31	-0.43, 1.0	0.417
>12wkCOVID	0.18	-0.55, 0.92	0.624
**Fatigue**	-0.04	-0.57, 0.49	0.880
**Sex**			
Male	—	—	
Female	0.00	-0.65, 0.65	0.997
**Age**	0.04	-0.26, 0.34	0.794
**Effort * Reward**	-0.13	-0.49, 0.22	0.467
**Effort * Group_recoded**			
*Effort * noCOVID*	0.11	-0.50, 0.72	0.714
*Effort * >12wkCOVID*	0.13	-0.51, 0.76	0.697
**Reward * Group_recoded**			
*Reward * noCOVID*	0.08	-0.58, 0.73	0.822
*Reward * >12wkCOVID*	-0.50	-1.2, 0.18	0.148
**Effort * Fatigue**	0.05	-0.41, 0.51	0.835
**Reward * Fatigue**	0.00	-0.49, 0.49	0.988
**Group_recoded * Fatigue**			
*noCOVID * Fatigue*	-0.24	-1.0, 0.53	0.545
*>12wkCOVID * Fatigue*	0.00	-0.70, 0.69	0.992
**Effort * Reward * Group_recoded**			
*Effort * Reward * noCOVID*	-0.15	-0.64, 0.33	0.531
*Effort * Reward * >12wkCOVID*	-0.07	-0.57, 0.43	0.772
**Effort * Reward * Fatigue**	0.11	-0.25, 0.47	0.541
**Effort * Group_recoded * Fatigue**			
*Effort * noCOVID * Fatigue*	0.11	-0.55, 0.76	0.751
*Effort * >12wkCOVID * Fatigue*	0.14	-0.47, 0.75	0.649
**Reward * Group_recoded * Fatigue**			
*Reward * noCOVID * Fatigue*	-0.02	-0.72, 0.69	0.962
*Reward * >12wkCOVID * Fatigue*	-0.75	-1.4, -0.11	**0.022**
**Effort * Reward * Group_recoded * Fatigue**			
*Effort * Reward * noCOVID * Fatigue*	-0.20	-0.72, 0.32	0.451
*Effort * Reward * >12wkCOVID * Fatigue*	0.19	-0.29, 0.67	0.435

[1]OR = Odds Ratio, CI = Confidence Interval

Table S5c: To test whether the associations of fatigue with reward and effort and differed between the groups (model 3a, ref: noCOVID)

**Characteristic**	**log(OR)**[1]	**95% CI**[1]	**p-value**
**Effort**	2.2	1.8, 2.6	**<0.001**
**Reward**	3.0	2.6, 3.4	**<0.001**
**group**			
noCOVID	—	—	
<4wkCOVID	-0.45	-1.1, 0.24	0.205
>12wkCOVID	-0.31	-1.0, 0.40	0.394
**Depression**	-0.04	-0.45, 0.36	0.836
**Sex**			
Male	—	—	
Female	0.02	-0.63, 0.67	0.951
**Age**	0.07	-0.23, 0.36	0.650
**Effort * Reward**	-0.26	-0.55, 0.03	0.083
**Effort * Group**			
*Effort * <4wkCOVID*	-0.11	-0.68, 0.45	0.694
*Effort * >12wkCOVID*	0.11	-0.42, 0.64	0.682
**Reward * Group**			
*Reward * <4wkCOVID*	-0.07	-0.70, 0.56	0.829
*Reward * >12wkCOVID*	-1.0	-1.6, -0.41	**<0.001**
**Effort * Depression**	0.32	-0.03, 0.66	0.075
**Reward * Depression**	-0.04	-0.43, 0.35	0.841
**Group* Depression**			
*<4wkCOVID * Depression*	-0.08	-0.74, 0.57	0.802
*>12wkCOVID * Depression*	0.09	-0.52, 0.71	0.766
**Effort * Reward * Group**			
*Effort * Reward * <4wkCOVID*	0.11	-0.34, 0.55	0.643
*Effort * Reward * >12wkCOVID*	0.14	-0.28, 0.55	0.520
**Effort * Reward * Depression**	-0.02	-0.30, 0.25	0.878
**Effort * Group * Depression**			
*Effort * <4wkCOVID * Depression*	-0.50	-1.1, 0.07	0.086
*Effort * >12wkCOVID * Depression*	-0.11	-0.64, 0.43	0.698
**Reward * Group * Depression**			
*Reward * <4wkCOVID * Depression*	0.13	-0.50, 0.76	0.681
*Reward * >12wkCOVID * Depression*	-0.12	-0.72, 0.47	0.681
**Effort * Reward * Group * Depression**			
*Effort * Reward * <4wkCOVID * Depression*	0.09	-0.35, 0.53	0.690
*Effort * Reward * >12wkCOVID * Depression*	0.51	0.09, 0.93	**0.017**

[1]OR = Odds Ratio, CI = Confidence Interval

Table S5d: To test whether the associations of depressive mood with reward and effort differed between the groups (model 3b, ref: COVID <4 weeks)

**Characteristic**	**log(OR)**[1]	**95% CI**[1]	**p-value**
**Effort**	2.1	1.6, 2.5	**<0.001**
**Reward**	3.0	2.5, 3.5	**<0.001**
**Group_recoded**			
<4wkCOVID	—	—	
noCOVID	0.45	-0.24, 1.1	0.205
>12wkCOVID	0.14	-0.55, 0.83	0.696
**Depression**	-0.13	-0.64, 0.39	0.631
**Sex**			
Male	—	—	
Female	0.02	-0.63, 0.67	0.951
**Age**	0.07	-0.23, 0.36	0.650
**Effort * Reward**	-0.15	-0.50, 0.20	0.395
**Effort * Group_recoded**			
*Effort * noCOVID*	0.11	-0.45, 0.68	0.694
*Effort * >12wkCOVID*	0.23	-0.36, 0.81	0.450
**Reward * Group_recoded**			
*Reward * noCOVID*	0.07	-0.57, 0.70	0.830
*Reward * >12wkCOVID*	-0.93	-1.6, -0.28	**0.005**
**Effort * Depression**	-0.18	-0.63, 0.27	0.432
**Reward * Depression**	0.09	-0.40, 0.59	0.714
**Group_recoded * Depression**			
*noCOVID * Depression*	0.08	-0.57, 0.74	0.801
*>12wkCOVID * Depression*	0.18	-0.52, 0.87	0.617
**Effort * Reward * Group_recoded**			
*Effort * Reward * noCOVID*	-0.11	-0.55, 0.34	0.643
*Effort * Reward * >12wkCOVID*	0.03	-0.43, 0.49	0.895
**Effort * Reward * Depression**	0.07	-0.28, 0.42	0.699
**Effort * Group_recoded * Depression**			
*Effort * noCOVID * Depression*	0.50	-0.07, 1.1	0.087
*Effort * >12wkCOVID * Depression*	0.39	-0.22, 0.99	0.207
**Reward * Group_recoded * Depression**			
*Reward * noCOVID * Depression*	-0.13	-0.76, 0.50	0.681
*Reward * >12wkCOVID * Depression*	-0.26	-0.92, 0.41	0.451
**Effort * Reward * Group_recoded * Depression**			
*Effort * Reward * noCOVID * Depression*	-0.09	-0.53, 0.35	0.690
*Effort * Reward * >12wkCOVID * Depression*	0.42	-0.05, 0.89	0.081

[1]OR = Odds Ratio, CI = Confidence Interval

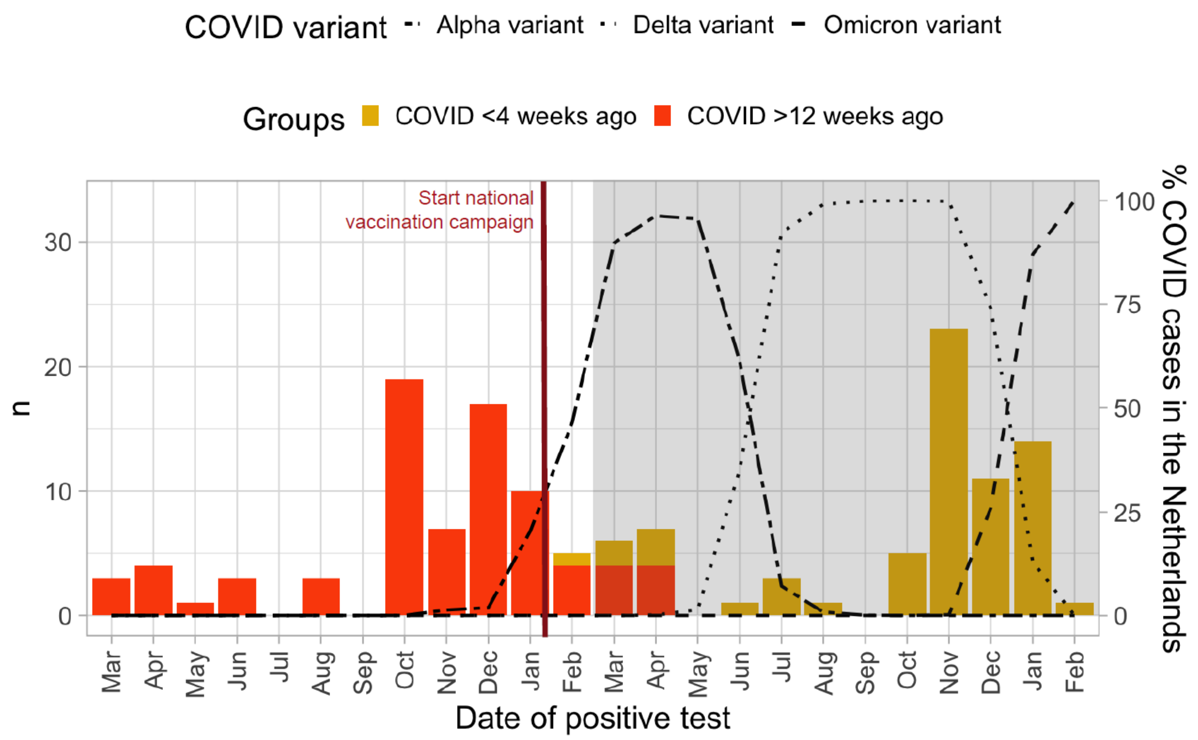

Supplementary Figure S1: Overview of inclusions of participants who had COVID-19 < 4 weeks and > 12 weeks ago, and active COVID-19 variants at that moment in the Netherlands (COVID-19 | RIVM, n.d.). The inclusion period is indicated by the grey area.

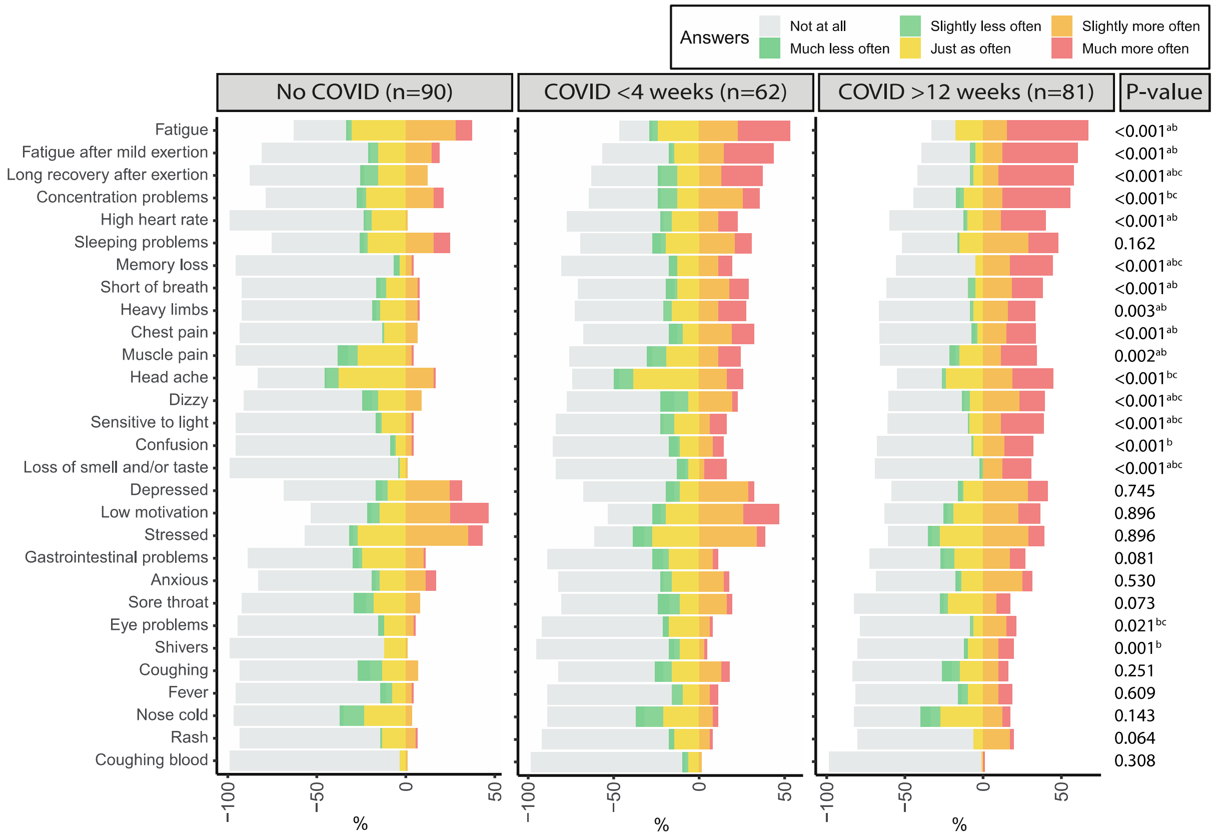

Supplementary Figure S2: Physical and psychological symptoms at the moment of participation compared to before the pandemic.

P-values for differences between groups were determined by performing chi-square tests.

[a]No COVID-19 group differs significantly from the COVID-19 < 4 weeks group (P < 0.05). [b]No COVID-19 group differs significantly from the COVID-19 > 12 weeks group (P < 0.05). [c]COVID-19 < 4 weeks group differs significantly from the COVID-19 >12 weeks group (P < 0.05).

Supplementary material for: Scholing, et al. Greater fatigue is more strongly associated with reduced reward sensitivity in the long-term phase of coronavirus disease (COVID-19) than in the early phase.

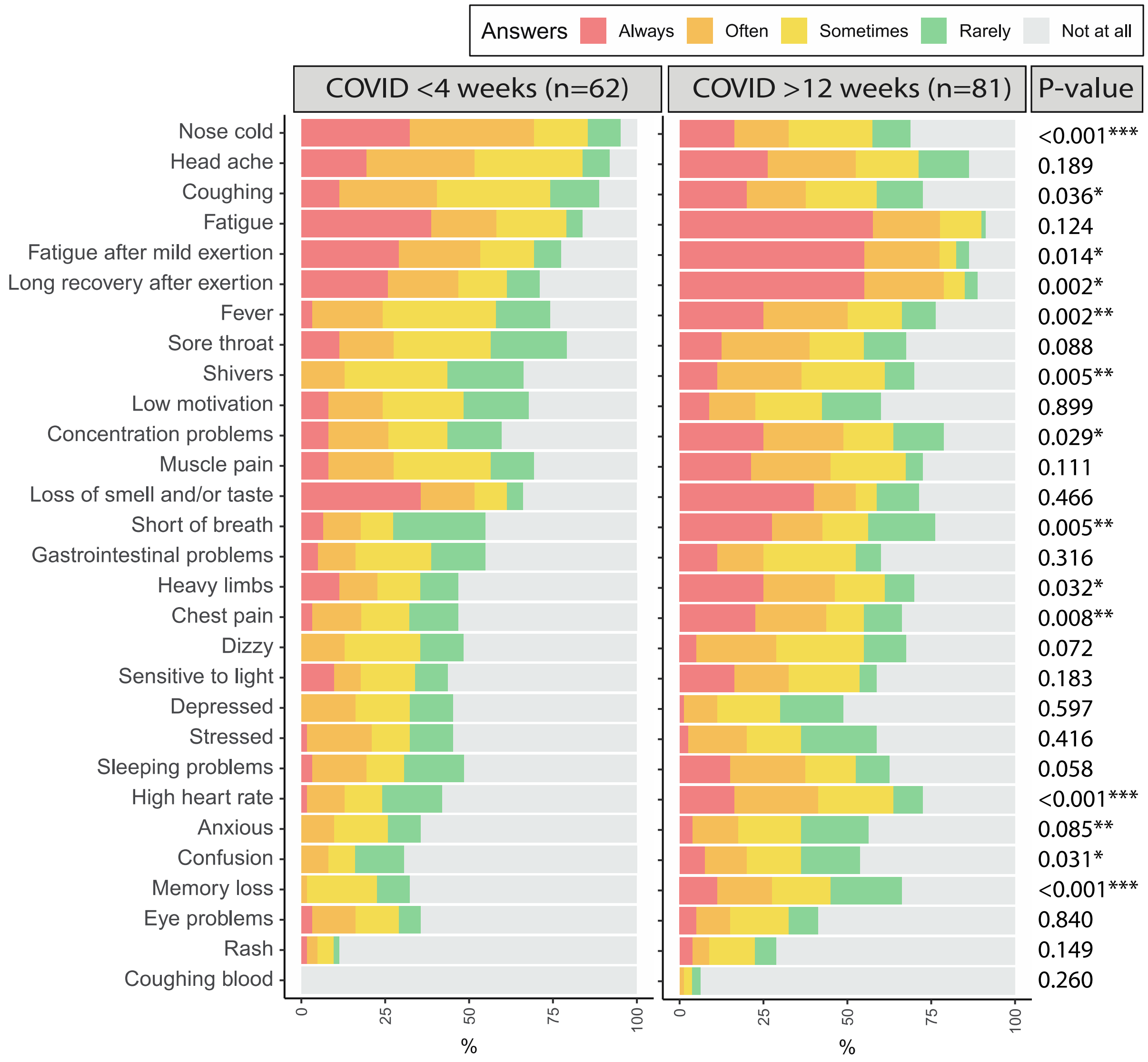

Supplementary Figure S3: Physical and psychological symptoms in the first 2 weeks of the acute infection compared to before the pandemic.

P-values for differences between groups were determined by performing chi-square tests.

*P < 0.05, **P < 0.01, ***P < 0.001

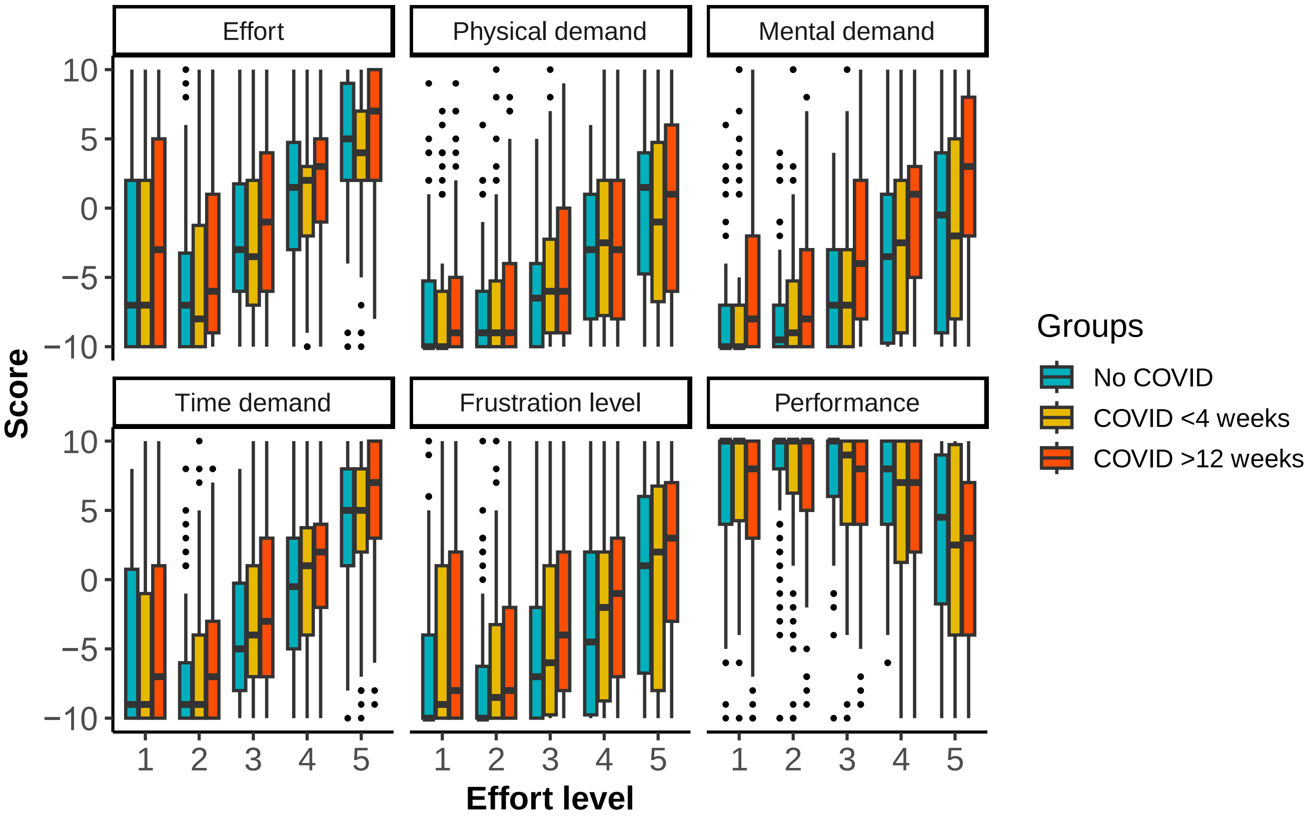

Supplementary Figure S4: NASA task load according to effort level of the effort-based decision making task.

Differences in NASA scores for increasing effort levels and between groups were tested using two-way ANOVAs. All NASA scores differed significantly with increasing effort levels (all $P < 0.001$), but did not differ between groups (all $P > 0.05$).

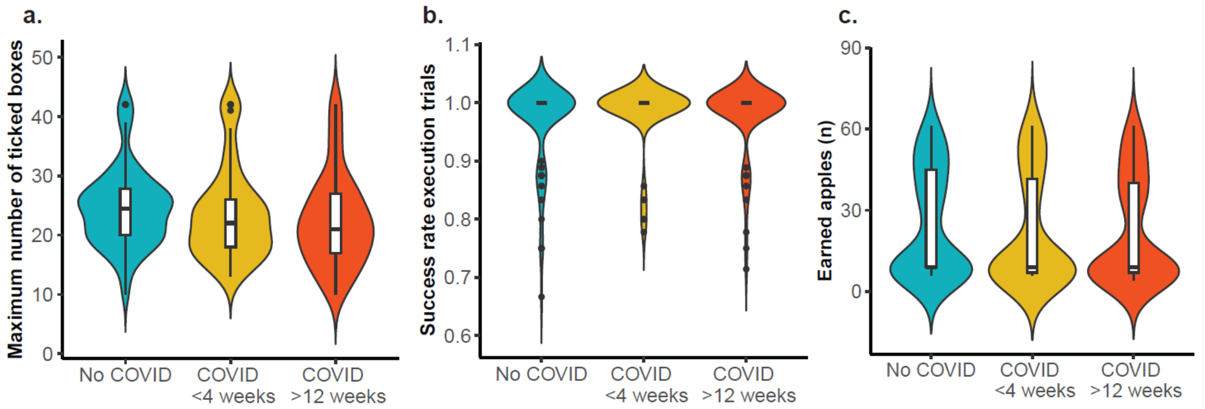

Supplementary Figure S5: Maximum number of ticked boxes, success rate of the execution trials and earned apples during the execution phase of the effort-based decision making task according to group. Differences between groups were tested by a one-way ANOVA with Tukey-HSD post hoc test.

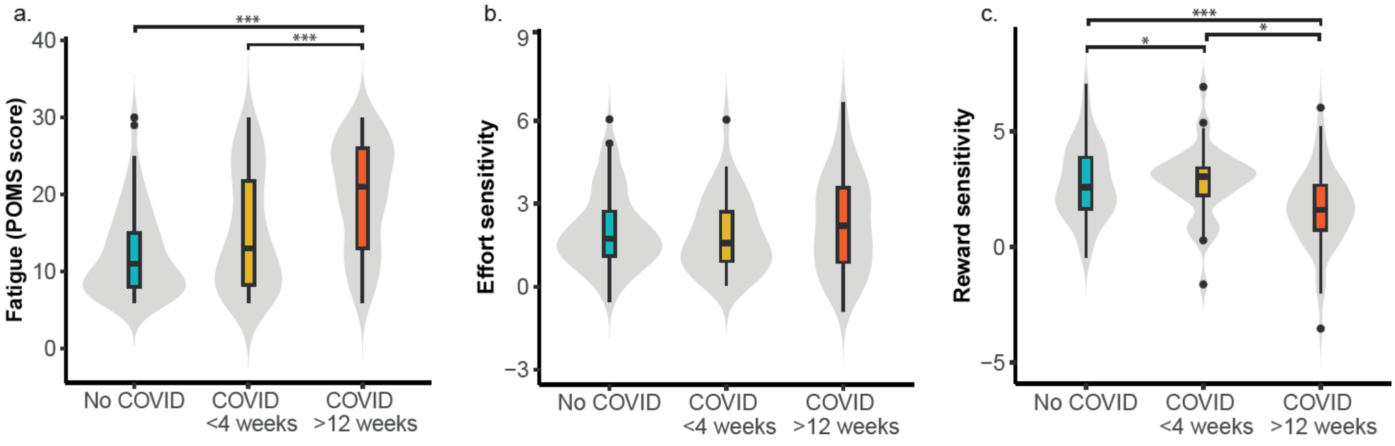

Supplementary Figure S6: Fatigue, effort sensitivity and reward sensitivity in a non-recovered subgroup (n = 195).

State fatigue (a) scores at moment of participation according to group and mean values of reward sensitivity (b) and effort sensitivity (c) in the non-recovered population. Regression coefficients for effort and reward were determined by mixed model binomial regression analysis.

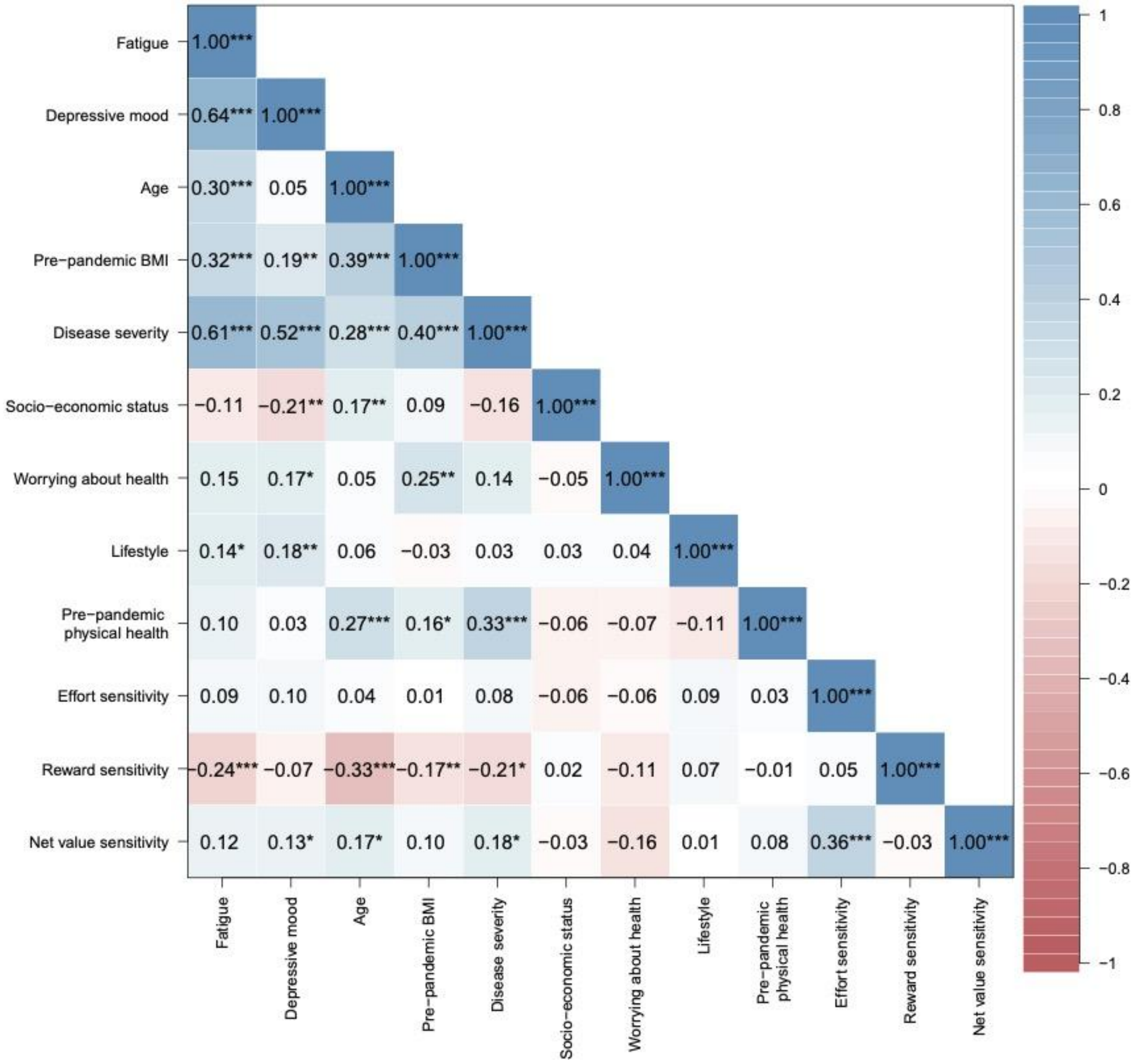

Supplementary Figure S7: Correlation matrix (Pearson correlations) of predictor and outcome variables across all three groups.

*P < 0.05, **P < 0.01, ***P < 0.001

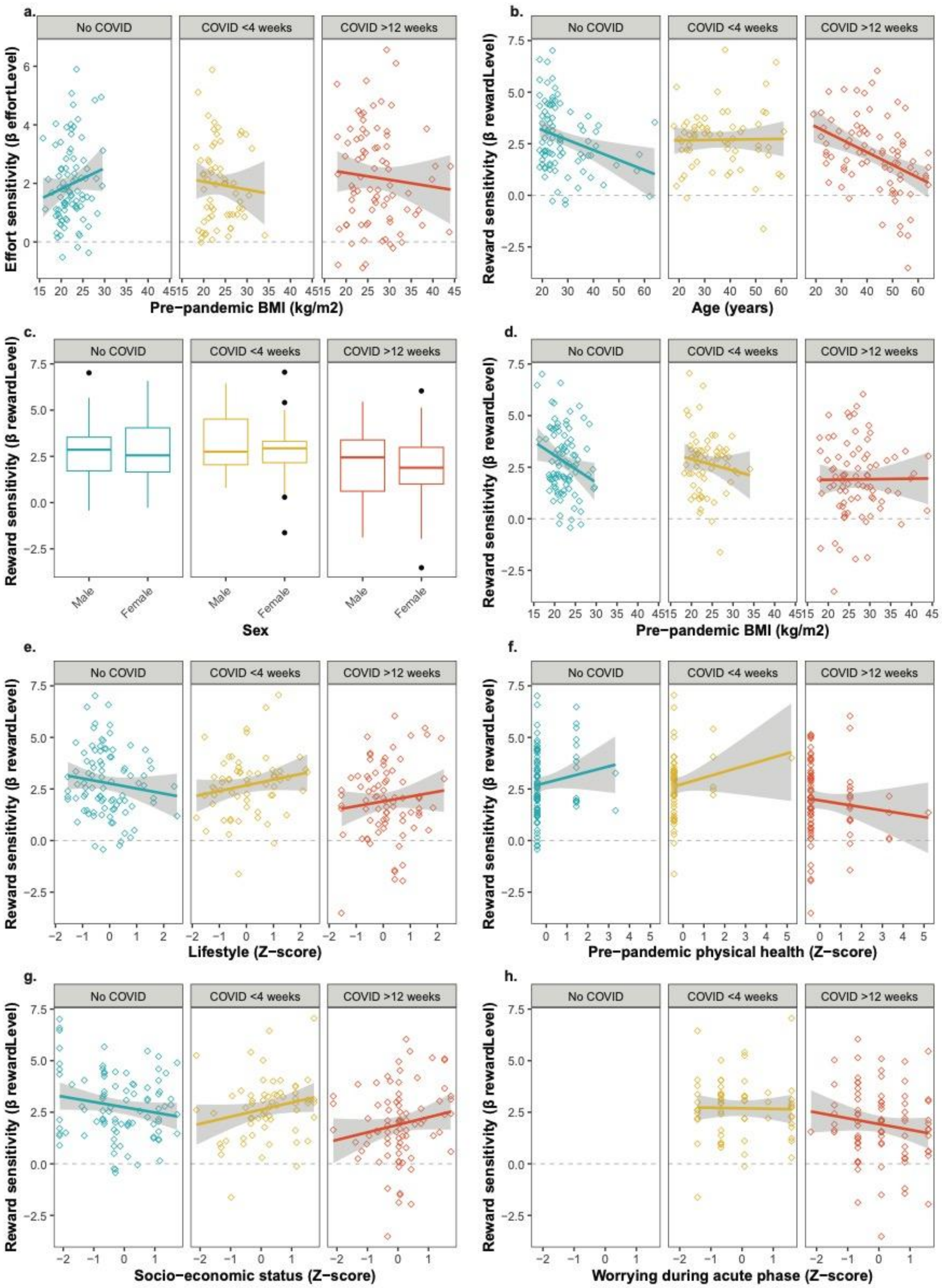

Supplementary Figure S8: Scatterplots of the associations of significant predictors with effort and/or reward sensitivity according to group.
