# Supplementary material for: Greater fatigue is more strongly associated with reduced reward sensitivity in the long-term phase of coronavirus disease (COVID-19) than in the early phase
Source: Brain Behav Immun Health. 2025 Jul 5;48:101056. doi: 10.1016/j.bbih.2025.101056 (PMC12275946; doi:10.1016/j.bbih.2025.101056)
Supplement: Multimedia component 2 [file mmc2.pdf]

Vermoeidheid en ziektegedrag na COVID-19

Waar gaat dit onderzoek over?

Veel mensen die COVID hebben of hebben gehad, hebben last van vermoeidheid en andere gezondheidsklachten. Bij sommige mensen houden deze klachten lang aan. Er is nog veel onduidelijk over deze vermoeidheid en hoe het verschilt van ‘gewone’ vermoeidheid. Met deze online studie hopen we daar meer inzicht in te krijgen.

Dit doen we door de gezondheid en welzijn van mensen die COVID hebben (gehad) te vergelijken met die van mensen die geen COVID hebben gehad. Daarnaast zullen we met een computerspel uw keuzegedrag meten. Dit geeft ons meer inzicht in hoe vermoeidheid na COVID verschilt van 'gewone' vermoeidheid. We hopen daarmee te kunnen bijdragen aan betere post-COVID gezondheidszorg.

Wie kan deelnemen?

U kunt deelnemen aan dit onderzoek als u:

- Op dit moment, of in de afgelopen 4 weken een bevestigde COVID-19 infectie heeft of heeft gehad **Of:**
- Meer dan 12 weken geleden een een bevestigde COVID-19 infectie heeft gehad

- Tussen de 18 en 65 jaar oud bent
- De Nederlandse taal kunt begrijpen

We zijn op zoek naar mensen met en zonder klachten. Het maakt dus niet uit of u veel, weinig of geen klachten heeft (gehad).

Aan deze studie zullen ongeveer 300 personen deelnemen.

Wat houdt deelname in?

- Vragenlijst: We vragen u om een vragenlijst in te vullen over uw gezondheid en over uw klachten tijdens uw COVID-infectie. Deze vragenlijsten zullen ongeveer 20 minuten duren.
- Computerspel: Daarnaast zullen we met een computerspel uw keuzegedrag onderzoeken. Dit spel zal ongeveer 15-20 minuten duren. Voor het computerspel is het belangrijk dat u via een laptop, desktop-computer of iPad/tablet deelneemt aan dit onderzoek, bij voorkeur met een muis.

Voor- en nadelen?

Er zijn geen voor- en nadelen van dit onderzoek, behalve dat het tijd kost om mee te doen.

Onder de deelnemers zullen wij **40 Bol.com-bonnen** verloten. Deze hebben tenminste een waarde van €5,-, maar bij het computerspel kunt u €0 tot €5,- extra verdienen. Mocht u willen meeloten voor de Bol.com-bon, dan kunt u na afloop een e-mailadres achterlaten. Dit is niet verplicht.

Vragen?

Dit onderzoek wordt uitgevoerd vanuit het Donders Instituut, Radboud Universiteit in Nijmegen door dr. Marieke van der Schaaf, dr. Esther Aarts, en Judith Scholing. Mocht u meer informatie willen of heeft u vragen over dit onderzoek, dan kunt u contact opnemen via het e-mailadres:

vermoeidheid-na-covid@donders.ru.nl

Bij deelname aan dit onderzoek moet u weten dat:

- Uw deelname is vrijwillig. U kunt op elk moment stoppen met het onderzoek.
- Uw gegevens die voor dit onderzoek worden verzameld zijn anoniem en worden gebruikt voor wetenschappelijk onderzoek. Er worden geen persoonsgegevens zoals uw naam of geboortedatum verzameld.
- Alleen als u dat zelf wilt kunt u een e-mailadres achterlaten zodat wij een voucher kunnen toesturen. Deze gegevens worden los van de andere studiegegevens bewaard. Ook kunt u aangeven of u benaderd mag worden voor vervolgonderzoek. Ook daarvoor geldt dat deelname vrijwillig is.
- Uw privacy wordt beschermd door de Algemene Verordening Gegevensbescherming (AVG).

☐ Ik bevestig dat ik bovenstaande informatie heb begrepen en wil graag deelnemen aan dit onderzoek.

Next

Heeft u COVID-19 gehad of denkt u dat u COVID-19 heeft gehad?

- ◯ Ja dit is bevestigd met een PCR test of sneltest (een swab-test)
- ◯ Ja dit is bevestigd met een antistoftest (een bloedtest)
- ◯ Ja maar dit is niet bevestigd met een test
- ◯ Nee
- ◯ Ik weet het niet

Indien u COVID-19 heeft gehad, hoelang geleden kreeg u voor het eerst klachten?

- ◯ Minder dan 4 weken geleden
- ◯ Tussen de 4 en 12 weken geleden
- ◯ Meer dan 12 weken geleden
- ◯ Ik heb geen COVID-19 gehad

Wat is uw leeftijd?

0

10 ◯ **100**

Hoe heeft u dit onderzoek gevonden?

- ◯ Via de Facebookgroep 'Corona patienten met langdurige klachten'
- ◯ Via de Facebookgroep 'Genezen van Corona'
- ◯ Via de Facebookgroep 'Reuk- en smaakverlies na Covid-19'
- ◯ Via een andere Facebook(groep)
- ◯ Via Twitter
- ◯ Via LinkedIn
- ◯ Via SONA
- ◯ Via Proefbunny
- ◯ Via vrienden/familie
- ◯ Anders, namelijk:

Next

≡

Vragenlijsten: Deel 1 van 7

Wat is uw biologische geslacht?

- ◯ Man
- ◯ Vrouw
- ◯ Anders

Wat is uw lengte (in cm)?

Wat was uw gewicht (in kg) voor maart 2020?

Wat is uw gewicht (in kg) op dit moment?

Welke etniciteit past het beste bij u?

- ◯ Nederlands/West-Europees
- ◯ Turks
- ◯ Marokkaans
- ◯ Surinaams
- ◯ Aziatisch
- ◯ Antilliaans
- ◯ Anders, namelijk:

Wat is uw hoogst afgeronde opleiding?

- ◯ Basisonderwijs
- ◯ VMBO of MAVO
- ◯ HAVO of VWO
- ◯ MBO
- ◯ HBO
- ◯ WO (bachelor)
- ◯ WO (master)

Hoeveel mensen, inclusief uzelf, wonen momenteel in uw huishouden?

- ◯ 1
- ◯ 2
- ◯ 3-4
- ◯ 5-6
- ◯ Anders, namelijk:

Hoeveel van hen zijn jonger dan 18 jaar?

Wat zijn de eerste 4 cijfers van uw postcode?

◯ Dat wil ik niet zeggen

Wat is momenteel uw werksituatie?

◯ Fulltime baan met een vast contract
◯ Fulltime baan met een tijdelijk contract
◯ Deeltijds baan met een vast contract
◯ Deeltijds baan met een tijdelijk contract
◯ Zelfstandig
◯ Werkend als freelancer
◯ Met ouderschapsverlof
◯ Met ziekteverlof (langdurig)
◯ Werkloos met een sociale uitkering
◯ Werkloos zonder sociale uitkering
◯ Voltijds studerend
◯ Gepensioneerd
◯ Anders, namelijk:

Wat is voor u het meest van toepassing?

◯ Ik werk vooral thuis
◯ Ik werk deels thuis en ga deels naar mijn werk
◯ Ik werk niet thuis
◯ Niet van toepassing

Hoeveel uur werkte u gemiddeld per week <u>voor maart 2020</u>?

◯ Ik werkte niet

Hoeveel uur werkte u gemiddeld per week <u>in de afgelopen 2 weken</u>?

◯ Ik werk niet

Indien uw werkuren zijn veranderd sinds maart 2020, wat was de reden hiervoor?

◯ Ik kan door mijn gezondheid niet meer zoveel werken als voor maart 2020
◯ Mijn werkzaamheden zijn door de coronamaatregelen veranderd
◯ Ik ben veranderd van baan
◯ Niet van toepassing
◯ Anders, namelijk:

Welke van de volgende opties beschrijft uw huidige beroep(en) het best?

☐ In opleiding
☐ Onderwijs of onderzoek
☐ Kunst

- [ ] Amusement
- [ ] Sport en media
- [ ] Gezondheidszorg
- [ ] Eerstelijns hulpverlener (paramedicus/brandweerman/politie)
- [ ] Militair
- [ ] Ambtenaren
- [ ] Politiek
- [ ] Financiën en economie
- [ ] Industrie
- [ ] Verkoop en diensten (incl. restaurants en bars)
- [ ] Transport (goederen en mensen)
- [ ] Installatie
- [ ] Onderhoud
- [ ] Reiniging en reparaties
- [ ] Bouw
- [ ] Landbouw
- [ ] Visserij en bosbouw
- [ ] Kantoor- en administratieve ondersteuning
- [ ] Ik werk momenteel niet
- [ ] Anders, namelijk:

Wat is het jaarlijks bruto inkomen van uw huishouden?

- ◯ 0 tot 5.000 euro
- ◯ 5.000 tot 10.000 euro
- ◯ 10.000 tot 15.000 euro
- ◯ 15.000 tot 25.000 euro
- ◯ 25.000 tot 50.000 euro
- ◯ 50.000 tot 75.000 euro
- ◯ 75.000 tot 100.000 euro
- ◯ 100.000 tot 125.000 euro
- ◯ 125.000 tot 150.000 euro
- ◯ 150.000 tot 175.000 euro
- ◯ 175.000 tot 200.000 euro
- ◯ Meer dan 200.000 euro

Bent u gevaccineerd tegen COVID-19?

- ◯ Nee
- ◯ Ja met het Pfizer vaccin
- ◯ Ja met het Moderna vaccin
- ◯ Ja met het AstraZeneca vaccin
- ◯ Ja met het Janssen vaccin
- ◯ Ja maar ik weet niet met welk vaccin
- ◯ Ja met een ander of verschillende typen vaccins, namelijk:

Indien u gevaccineerd bent, hoeveel keer bent u gevaccineerd?

- ◯ 1 keer
- ◯ 2 keer

- ◯ 3 keer
- ◯ 4 keer
- ◯ Ik ben niet gevaccineerd

Indien u gevaccineerd bent, op welke datum ontving u uw eerste vaccinatie?

- ◯ Ik ben niet gevaccineerd

Indien u gevaccineerd bent, op welke datum ontving u uw laatste vaccinatie?

- ◯ Ik ben niet gevaccineerd

Next

Vragenlijsten: Deel 2 van 7

Op welke datum was uw positieve coronatest afgenomen?

23 | May | 2024

Op welke datum kreeg u voor het eerst klachten?
Indien u geen klachten had kunt u de datum laten staan op de datum van vandaag (bijvoorbeeld als u later positief testte op antistoffen).

23 | May | 2024

Welke klachten heeft/had u in de eerste 2 weken van uw COVID-19 infectie?

	Niet	Zelden	Soms	Vaak	Voort-durend
Hoesten	○	○	○	○	○
Neusverkoudheid	○	○	○	○	○
Keelpijn	○	○	○	○	○
Koorts of verhoging	○	○	○	○	○
Spierpijn/gewrichtspijn	○	○	○	○	○
Zware armen of benen	○	○	○	○	○
Vermoeidheid	○	○	○	○	○
Hoofdpijn	○	○	○	○	○
Rillingen	○	○	○	○	○
Benauwdheid	○	○	○	○	○
Verhoogde hartslag	○	○	○	○	○
Meer vermoeidheid na milde inspanning	○	○	○	○	○
Langer herstel nodig na inspanning	○	○	○	○	○
Pijn of druk op de borst	○	○	○	○	○
Bloed ophoesten	○	○	○	○	○
Verlies of verandering van reuk en/of smaak	○	○	○	○	○
Huiduitslag	○	○	○	○	○
Maag-darm problemen (buikpijn, misselijkheid, diarree)	○	○	○	○	○
Rode ogen/ oogpijn	○	○	○	○	○
Verwardheid	○	○	○	○	○
Geheugenverlies	○	○	○	○	○
Concentratieproblemen	○	○	○	○	○
Verhoogde gevoeligheid voor licht en geluid	○	○	○	○	○
Duizeligheid	○	○	○	○	○

Slaapproblemen	◯	◯	◯	◯	◯
Depressieve gevoelens	◯	◯	◯	◯	◯
Angstige gevoelens	◯	◯	◯	◯	◯
Stress	◯	◯	◯	◯	◯
Verminderde motivatie	◯	◯	◯	◯	◯

Heeft u nog andere klachten gehad in de eerste 2 weken van uw COVID-19 infectie?

◯ Ik had geen andere klachten.

Hoe heeft u uw klachten ervaren?

◯ Ik ben veel zieker geworden dan ik van tevoren had gedacht
◯ Ik ben even ziek geworden als ik van te voren had gedacht
◯ Ik ben minder ziek geworden dan ik van te voren had gedacht

Hoe vaak maakte u zich zorgen om uw gezondheid?

	Niet	Zelden	Soms	Vaak	Voort-durend
Voor maart 2020	◯	◯	◯	◯	◯
In de eerste 2 weken van uw COVID-infectie	◯	◯	◯	◯	◯
In de afgelopen 2 weken	◯	◯	◯	◯	◯

Heeft u medische hulp gehad of geraadpleegd?

◯ Nee
◯ Via de huisarts
◯ Ik heb de spoedeisende hulp bezocht
◯ Ik ben opgenomen geweest op de verpleegafdeling in het ziekenhuis
◯ Ik ben opgenomen geweest op de intensive care

Indien van toepassing, hoeveel dagen bent u opgenomen geweest?

◯ Niet van toepassing

Heeft u zuurstof of beademing gehad?

◯ Ik heb extra zuurstof gehad
◯ Ik ben beademd geweest
◯ Nee

Hoe lang duurde het voordat u hersteld was?

◯ <2 weken
◯ 3-4 weken
◯ 5-10 weken
◯ 10+ weken

- ◯ Ik ben (nog) niet volledig hersteld
- ◯ Ik had geen klachten

Het herstel duurde...

- ◯ Korter dan ik had verwacht
- ◯ Ongeveer even lang als ik had verwacht
- ◯ Langer dan ik had verwacht
- ◯ Niet van toepassing

Voelde u zich begrepen en ondersteund door de artsen die u behandelden?

- ◯ Helemaal niet
- ◯ Enigszins
- ◯ Nogal
- ◯ Veel
- ◯ Heel veel
- ◯ Niet van toepassing

Heeft u nazorg gehad? Zo ja, wat voor?

- ☐ Fysiotherapie
- ☐ Ergotherapie
- ☐ Bij de psycholoog
- ☐ Door een maatschappelijk werker
- ☐ Ik ben doorverwezen naar de long-poli
- ☐ Ik ben doorverwezen naar de neurologie
- ☐ Ik ben doorverwezen naar de psychiatrie
- ☐ Nee
- ☐ Anders, namelijk:

Hoe tevreden bent u met de nazorg na COVID-19?

- ◯ Heel erg tevreden
- ◯ Enigszins tevreden
- ◯ Niet tevreden
- ◯ Totaal niet tevreden
- ◯ Niet van toepassing

Heeft u zich ziekgemeld of uw werk niet kunnen doen door uw COVID-19 klachten? Zo ja, hoe lang?

- ◯ 1-2 weken
- ◯ 2-5 weken
- ◯ >5 weken
- ◯ Nee

Welke klachten had u <u>in de afgelopen 2 weken</u>?
Het gaat hier bij om alle corona en niet-corona klachten die u had.

- Als u de klacht **niet** had, kruis dan het vakje **'niet'** aan.
- Als u de klacht **wel** had, geef dan aan of u deze klacht de laatste tijd vaker, even vaak of minder vaak heeft **dan voor maart 2020**.

Voorbeeld 1: u had de afgelopen 2 weken last van hoofdpijn, maar dat had u ook wel eens voor maart

2020. Dan kruist u 'even vaak' aan.
Voorbeeld 2: u had in de afgelopen 2 weken last van hoofdpijn en u heeft het gevoel dat u de laatste tijd veel vaker hoofdpijn heeft dan voor maart 2020. Dan kruist u 'Veel vaker' aan.

	**Niet**	**Veel minder vaak**	**Iets minder vaak**	**Even vaak**	**Iets vaker**	**Veel vaker**
Hoesten	○	○	○	○	○	○
Neusverkoudheid	○	○	○	○	○	○
Keelpijn	○	○	○	○	○	○
Koorts of verhoging	○	○	○	○	○	○
Spierpijn/gewrichtspijn	○	○	○	○	○	○
Zware armen of benen	○	○	○	○	○	○
Vermoeidheid	○	○	○	○	○	○
Hoofdpijn	○	○	○	○	○	○
Rillingen	○	○	○	○	○	○
Benauwdheid	○	○	○	○	○	○
Verhoogde hartslag	○	○	○	○	○	○
Meer vermoeidheid na milde inspanning	○	○	○	○	○	○
Langer herstel nodig na inspanning	○	○	○	○	○	○
Pijn of druk op de borst	○	○	○	○	○	○
Bloed ophoesten	○	○	○	○	○	○
Verlies of verandering van reuk en/of smaak	○	○	○	○	○	○
Huiduitslag	○	○	○	○	○	○
Maag-darm problemen (buikpijn, misselijkheid, diarree)	○	○	○	○	○	○
Rode ogen/ oogpijn	○	○	○	○	○	○
Verwardheid	○	○	○	○	○	○
Geheugenverlies	○	○	○	○	○	○
Concentratieproblemen	○	○	○	○	○	○
Verhoogde gevoeligheid voor licht en geluid	○	○	○	○	○	○
Duizeligheid	○	○	○	○	○	○
Slaapproblemen	○	○	○	○	○	○
Depressieve gevoelens	○	○	○	○	○	○
Angstige gevoelens	○	○	○	○	○	○
Stress	○	○	○	○	○	○
Verminderde motivatie	○	○	○	○	○	○

Heeft u nog andere nieuwe klachten gekregen sinds maart 2020?
Zo ja, welke?

◯ Ik heb geen andere nieuwe klachten gehad.

Indien uw klachten zijn veranderd sinds maart 2020, wat is volgens u hiervan de reden?

◯ Vooral door de lockdownmaatregelen
◯ Vooral door mijn COVID-19 infectie
◯ Door mijn COVID-19 infectie EN de lockdown maatregelen
◯ Mijn klachten zijn niet veranderd
◯ Anders, namelijk:

Indien u last heeft van vermoeidheid, zou u kunnen omschrijven hoe u uw vermoeidheid ervaart?

◯ Ik heb geen last (meer) van vermoeidheid

Next

Vragenlijsten: Deel 2 van 7

Hoe zeker bent u dat u geen COVID-19 heeft gehad?

- Heel zeker
- Redelijk zeker
- Een beetje zeker
- Niet zeker
- Helemaal niet zeker

Heeft u zich wel eens laten testen op COVID-19 als u klachten had?

- Ja elke keer als ik klachten had. De test was negatief.
- Soms. De test was negatief.
- Nee. Ik heb mij niet laten testen als ik klachten had.
- Niet van toepassing. Ik heb geen klachten gehad.

Heeft u zich wel eens laten testen op COVID-19 om een andere reden (bijvoorbeeld na nauw contact met een positief getest persoon, of na terugkomst uit een risicogebied)?

- Ja elke keer wanneer dit het geval was. De test was negatief.
- Soms. De test was negatief.
- Nee. Ik heb mij niet laten testen als dit het geval was.
- Niet van toepassing. Ik heb geen nauw contact gehad met een positief getest persoon en ben niet in een risicogebied geweest.

Heeft u zich wel eens laten testen op COVID-19 zonder directe reden?

- Ja. De test was negatief.
- Nee

Heeft u zich wel eens laten testen op antistoffen voor COVID-19 (door een bloedtest)?

- Ja. De test was negatief. Ik had geen antistoffen.
- Nee

Welke klachten had u in de afgelopen 2 weken?

- Als u de klacht **niet** had, kruis dan het vakje **'niet'** aan.
- Als u de klacht **wel** had, geef dan aan of u deze klacht de laatste tijd vaker, even vaak of minder vaak heeft **dan voor maart 2020**.

Voorbeeld 1: u had de afgelopen 2 weken last van hoofdpijn, maar dat had u ook wel eens voor maart 2020. Dan kruist u 'even vaak' aan.
Voorbeeld 2: u had in de afgelopen 2 weken last van hoofdpijn en u heeft het gevoel dat u de laatste tijd veel vaker hoofdpijn heeft dan voor maart 2020. Dan kruist u 'Veel vaker' aan.

	Niet	Veel minder vaak	Iets minder vaak	Even vaak	Iets vaker	Veel vaker
Hoesten	○	○	○	○	○	○
Neusverkoudheid	○	○	○	○	○	○

Keelpijn	○	○	○	○	○	○
Koorts of verhoging	○	○	○	○	○	○
Spierpijn/gewrichtspijn	○	○	○	○	○	○
Zware armen of benen	○	○	○	○	○	○
Vermoeidheid	○	○	○	○	○	○
Hoofdpijn	○	○	○	○	○	○
Rillingen	○	○	○	○	○	○
Benauwdheid	○	○	○	○	○	○
Verhoogde hartslag	○	○	○	○	○	○
Meer vermoeidheid na milde inspanning	○	○	○	○	○	○
Langer herstel nodig na inspanning	○	○	○	○	○	○
Pijn of druk op de borst	○	○	○	○	○	○
Bloed ophoesten	○	○	○	○	○	○
Verlies of verandering van reuk en/of smaak	○	○	○	○	○	○
Huiduitslag	○	○	○	○	○	○
Maag-darm problemen (buikpijn, misselijkheid, diarree)	○	○	○	○	○	○
Rode ogen/ oogpijn	○	○	○	○	○	○
Verwardheid	○	○	○	○	○	○
Geheugenverlies	○	○	○	○	○	○
Concentratieproblemen	○	○	○	○	○	○
Verhoogde gevoeligheid voor licht en geluid	○	○	○	○	○	○
Duizeligheid	○	○	○	○	○	○
Slaapproblemen	○	○	○	○	○	○
Depressieve gevoelens	○	○	○	○	○	○
Angstige gevoelens	○	○	○	○	○	○
Stress	○	○	○	○	○	○
Verminderde motivatie	○	○	○	○	○	○

Indien uw klachten zijn veranderd sinds maart 2020, wat is volgens u hiervan de reden?

○ Vooral door de lockdownmaatregelen
○ Mijn klachten zijn niet veranderd
○ Door een andere reden, namelijk:

Indien u last heeft van vermoeidheid, zou u kunnen omschrijven hoe u uw vermoeidheid ervaart?

○ Ik heb geen last van vermoeidheid

Next

Vragenlijsten: Deel 3 van 7

We willen u vragen de volgende vragen twee keer te beantwoorden: Over hoe uw situatie was voor maart 2020, en over hoe uw situatie in de afgelopen 2 weken was.

Hoeveel uur per week deed u een activiteit/sport waarbij u zich erg inspande (waarbij u buiten adem raakt)?

	Nooit	1-2 uur	2-3 uur	4-5 uur	meer dan 5 uur
Voor maart 2020	◯	◯	◯	◯	◯
In de afgelopen 2 weken	◯	◯	◯	◯	◯

Hoeveel uur per week deed u een sport/activiteit waarbij u zich matig inspande (waarbij praten mogelijk blijft, zoals wandelen en fietsen)?

	Nooit	1-2 uur	2-3 uur	4-5 uur	meer dan 5 uur
Voor maart 2020	◯	◯	◯	◯	◯
In de afgelopen 2 weken	◯	◯	◯	◯	◯

Rookt(e) u?

	Nee	Af en toe	Ja (bijna) dagelijks
Voor maart 2020	◯	◯	◯
In de afgelopen 2 weken	◯	◯	◯

Hoe vaak dronk u alcohol voor maart 2020?

- ◯ Nooit
- ◯ Maandelijks of minder
- ◯ 2-4 keer per maand
- ◯ 2-3 keer per week
- ◯ 4 of meer keer per week

Hoe vaak dronk u alcohol in de afgelopen 4 weken?

- ◯ Nooit
- ◯ Maandelijks of minder
- ◯ 2-4 keer per maand
- ◯ 2-3 keer per week
- ◯ 4 of meer keer per week

Had u een van de volgende chronische aandoeningen voor maart 2020?

- ☐ Diabetes type I
- ☐ Diabetes type II
- ☐ Hoge bloeddruk
- ☐ Chronische hartproblemen
- ☐ Chronische luchtweg of longproblemen

≡

- [ ] Kanker
- [ ] Auto-immuunziekte
- [ ] Ernstige leverziekte
- [ ] Nierziekte
- [ ] Hoge cholesterol
- [ ] Nee
- [ ] Anders, namelijk:

Heeft u een van de volgende chronische aandoeningen op dit moment (of nog steeds)?

- [ ] Diabetes type I
- [ ] Diabetes type II
- [ ] Hoge bloeddruk
- [ ] Chronische hartproblemen
- [ ] Chronische luchtweg of longproblemen
- [ ] Kanker
- [ ] Auto-immuunziekte
- [ ] Ernstige leverziekte
- [ ] Nierziekte
- [ ] Hoge cholesterol
- [ ] Nee
- [ ] Anders, namelijk:

Gebruikte u medicatie (die door een arts werd voorgeschreven, of vrij verkrijgbare medicatie) voor maart 2020?

○ Nee
○ Ja, namelijk:

Gebruikte u medicatie (die door een arts werd voorgeschreven, of vrij verkrijgbare medicatie) in de afgelopen 2 weken?

○ Nee
○ Ja, namelijk:

Next

Vragenlijsten: Deel 4 van 7

Hieronder vindt u enkele situaties waarin mensen zich kunnen bevinden **door de huidige corona pandemie en/of lockdown maatregelen**. Kunt u aangeven of u in de afgelopen twee weken onderstaande situaties heeft ervaren door de corona pandemie en/of maatregelen, en hoe hinderlijk dit voor u is/was?

Het hebben van corona symptomen, of symptomen die gerelateerd kunnen zijn aan corona.

- ◯ Deze situatie deed zich niet voor
- ◯ Niet hinderlijk
- ◯ Nauwelijks hinderlijk
- ◯ Enigszins hinderlijk
- ◯ Behoorlijk hinderlijk
- ◯ Erg hinderlijk

Corona symptomen, of symptomen die gerelateerd kunnen zijn aan corona bij gezinsleden, vrienden, dierbaren of collega's.

- ◯ Deze situatie deed zich niet voor
- ◯ Niet hinderlijk
- ◯ Nauwelijks hinderlijk
- ◯ Enigszins hinderlijk
- ◯ Behoorlijk hinderlijk
- ◯ Erg hinderlijk

Verhoogd risico op een infectie (bijvoorbeeld op werk, of in de supermarkt).

- ◯ Deze situatie deed zich niet voor
- ◯ Niet hinderlijk
- ◯ Nauwelijks hinderlijk
- ◯ Enigszins hinderlijk
- ◯ Behoorlijk hinderlijk
- ◯ Erg hinderlijk

Een verhoogd risico op een ernstig verloop van de ziekte bij een corona besmetting (behoren tot een zogenaamde risicogroep).

- ◯ Deze situatie deed zich niet voor
- ◯ Niet hinderlijk
- ◯ Nauwelijks hinderlijk
- ◯ Enigszins hinderlijk
- ◯ Behoorlijk hinderlijk
- ◯ Erg hinderlijk

Familie, vrienden, of dierbaren met een verhoogd risico op een ernstig ziekteverloop indien ze besmet raken (behoren tot een risicogroep).

- ◯ Deze situatie deed zich niet voor
- ◯ Niet hinderlijk
- ◯ Nauwelijks hinderlijk
- ◯ Enigszins hinderlijk

- ◯ Behoorlijk hinderlijk
- ◯ Erg hinderlijk

Problemen bij toegang tot gezondheidszorg, medicatie of sanitaire voorzieningen.

- ◯ Deze situatie deed zich niet voor
- ◯ Niet hinderlijk
- ◯ Nauwelijks hinderlijk
- ◯ Enigszins hinderlijk
- ◯ Behoorlijk hinderlijk
- ◯ Erg hinderlijk

Beperkt zijn in het verlaten van uw woning.

- ◯ Deze situatie deed zich niet voor
- ◯ Niet hinderlijk
- ◯ Nauwelijks hinderlijk
- ◯ Enigszins hinderlijk
- ◯ Behoorlijk hinderlijk
- ◯ Erg hinderlijk

Verlies van sociaal contact en sociale evenementen.

- ◯ Deze situatie deed zich niet voor
- ◯ Niet hinderlijk
- ◯ Nauwelijks hinderlijk
- ◯ Enigszins hinderlijk
- ◯ Behoorlijk hinderlijk
- ◯ Erg hinderlijk

Beperkt zijn bij het bezoeken van familie, vrienden of dierbaren in het ziekenhuis.

- ◯ Deze situatie deed zich niet voor
- ◯ Niet hinderlijk
- ◯ Nauwelijks hinderlijk
- ◯ Enigszins hinderlijk
- ◯ Behoorlijk hinderlijk
- ◯ Erg hinderlijk

Niet in staat zijn om een begrafenis of crematie van een familielid, vriend of andere naaste bij te wonen.

- ◯ Deze situatie deed zich niet voor
- ◯ Niet hinderlijk
- ◯ Nauwelijks hinderlijk
- ◯ Enigszins hinderlijk
- ◯ Behoorlijk hinderlijk
- ◯ Erg hinderlijk

Het hebben van familieleden, vrienden of dierbaren met een vitaal beroep (bijvoorbeeld zorg, politie of brandweer).

- ◯ Deze situatie deed zich niet voor
- ◯ Niet hinderlijk
- ◯ Nauwelijks hinderlijk
- ◯ Enigszins hinderlijk
- ◯ Behoorlijk hinderlijk
- ◯ Erg hinderlijk

Minder lichaamsbeweging dan normaal.

- ◯ Deze situatie deed zich niet voor
- ◯ Niet hinderlijk
- ◯ Nauwelijks hinderlijk
- ◯ Enigszins hinderlijk
- ◯ Behoorlijk hinderlijk
- ◯ Erg hinderlijk

Moeilijkheden om werk te combineren met kinderopvang.

- ◯ Deze situatie deed zich niet voor
- ◯ Niet hinderlijk
- ◯ Nauwelijks hinderlijk
- ◯ Enigszins hinderlijk
- ◯ Behoorlijk hinderlijk
- ◯ Erg hinderlijk

Spanningen in de thuissituatie of gezinsconflicten.

- ◯ Deze situatie deed zich niet voor
- ◯ Niet hinderlijk
- ◯ Nauwelijks hinderlijk
- ◯ Enigszins hinderlijk
- ◯ Behoorlijk hinderlijk
- ◯ Erg hinderlijk

Een verhoogde belasting door uw werk, of werk-gerelateerde obstakels.

- ◯ Deze situatie deed zich niet voor
- ◯ Niet hinderlijk
- ◯ Nauwelijks hinderlijk
- ◯ Enigszins hinderlijk
- ◯ Behoorlijk hinderlijk
- ◯ Erg hinderlijk

(Dreiging van) ontslag, insolventie van een privébedrijf, voor uzelf of iemand in uw huishouden.

- ◯ Deze situatie deed zich niet voor
- ◯ Niet hinderlijk
- ◯ Nauwelijks hinderlijk
- ◯ Enigszins hinderlijk
- ◯ Behoorlijk hinderlijk
- ◯ Erg hinderlijk

Problemen bij het verkrijgen van basisbehoeften en diensten.

- Deze situatie deed zich niet voor
- Niet hinderlijk
- Nauwelijks hinderlijk
- Enigszins hinderlijk
- Behoorlijk hinderlijk
- Erg hinderlijk

Corona-gerelateerde berichtgeving in de media.

- Deze situatie deed zich niet voor
- Niet hinderlijk
- Nauwelijks hinderlijk
- Enigszins hinderlijk
- Behoorlijk hinderlijk
- Erg hinderlijk

Anders (specificeer)

- Deze situatie deed zich niet voor
- Niet hinderlijk
- Nauwelijks hinderlijk
- Enigszins hinderlijk
- Behoorlijk hinderlijk
- Erg hinderlijk

Hieronder volgen enkele gebeurtenissen die mensen mogelijk ervaren **onafhankelijk van de huidige corona pandemie of maatregelen**, of gebeurtenissen die mogelijk zijn verergerd door de pandemie of maatregelen . Geef aan of u deze gebeurtenissen in de afgelopen twee weken heeft ervaren en hoe belastend deze voor u zijn/waren.

Financiële problemen.

- Deze situatie deed zich niet voor
- Niet hinderlijk
- Nauwelijks hinderlijk
- Enigszins hinderlijk
- Behoorlijk hinderlijk
- Erg hinderlijk

Conflicten of meningsverschillen in een familie-, sociale of professionele omgeving.

- Deze situatie deed zich niet voor
- Niet hinderlijk
- Nauwelijks hinderlijk
- Enigszins hinderlijk
- Behoorlijk hinderlijk
- Erg hinderlijk

Overlijden van een naaste.

- Deze situatie deed zich niet voor
- Niet hinderlijk
- Nauwelijks hinderlijk
- Enigszins hinderlijk
- Behoorlijk hinderlijk
- Erg hinderlijk

Scheiding van een naaste.

- Deze situatie deed zich niet voor
- Niet hinderlijk
- Nauwelijks hinderlijk
- Enigszins hinderlijk
- Behoorlijk hinderlijk
- Erg hinderlijk

Ernstige ziekte of psychische problemen bij uzelf of een naaste.

- Deze situatie deed zich niet voor
- Niet hinderlijk
- Nauwelijks hinderlijk
- Enigszins hinderlijk
- Behoorlijk hinderlijk
- Erg hinderlijk

Anders (specificeer)

- Deze situatie deed zich niet voor
- Niet hinderlijk
- Nauwelijks hinderlijk
- Enigszins hinderlijk
- Behoorlijk hinderlijk
- Erg hinderlijk

Next

Vragenlijsten: Deel 6 van 7

In dit deel van de vragenlijst wordt naar uw gezondheid gevraagd. Wanneer u twijfelt over het antwoord op een vraag, probeer dan het antwoord te geven dat het meest van toepassing is.

Wat vindt u, over het algemeen genomen, van uw gezondheid op dit moment?

- ◯ Uitstekend
- ◯ Zeer goed
- ◯ Goed
- ◯ Matig
- ◯ Slecht

In vergelijking met de periode voor maart 2020, hoe zou u nu uw gezondheid in het algemeen beoordelen?

- ◯ Veel beter dan voor maart 2020
- ◯ Iets beter dan voor maart 2020
- ◯ Ongeveer hetzelfde als voor maart 2020
- ◯ Iets slechter dan voor maart 2020
- ◯ Veel slechter dan voor maart 2020

De volgende vragen gaan over dagelijks bezigheden. Wordt u door uw gezondheid op dit moment beperkt bij deze bezigheden? Zo ja, in welke mate?

	Ernstig	Een beetje	Helemaal niet
Forse inspanning (zoals hardlopen, zware voorwerpen tillen, inspannend sporten)	◯	◯	◯
Matige inspanning (zoals het verplaatsen van een tafel, stofzuigen, fietsen)	◯	◯	◯
Tillen of boodschappen dragen	◯	◯	◯
Een paar trappen oplopen	◯	◯	◯
Een trap oplopen	◯	◯	◯
Buigen, knielen of bukken	◯	◯	◯
Meer dan een kilometer lopen	◯	◯	◯
Een halve kilometer lopen	◯	◯	◯

	Ernstig	Een beetje	Helemaal niet
Honderd meter lopen	◯	◯	◯

	Ernstig	Een beetje	Helemaal niet
Uzelf wassen of aankleden	◯	◯	◯

Had u, ten gevolge van uw lichamelijke gezondheid, de afgelopen 2 weken één van de volgende problemen bij uw werk of andere dagelijkse bezigheden?

	Ja	Nee
U heeft minder tijd kunnen besteden aan werk of andere bezigheden	◯	◯
U heeft minder bereikt dan u zou willen	◯	◯
U was beperkt in het soort werk of soort bezigheden	◯	◯
U had moeite met het werk of andere bezigheden (het kostte u bijvoorbeeld extra inspanning)	◯	◯

Had u, ten gevolge van een emotioneel probleem (bijvoorbeeld doordat u zich depressief of angstig voelde), de afgelopen 2 weken één van de volgende problemen bij uw werk of andere dagelijkse bezigheden?

	Ja	Nee
U heeft minder tijd kunnen besteden aan werk of andere bezigheden	◯	◯
U heeft minder bereikt dan u zou willen	◯	◯
U heeft het werk of andere bezigheden niet zo zorgvuldig gedaan als u gewend bent	◯	◯

In hoeverre heeft uw lichamelijke gezondheid of hebben uw emotionele problemen u de afgelopen 2 weken belemmerd in uw normale sociale bezigheden met gezin, vrienden, buren of anderen?

- ◯ Helemaal niet
- ◯ Enigszins
- ◯ Nogal
- ◯ Veel
- ◯ Heel erg veel

Hoeveel pijn had u de afgelopen 2 weken?

- ◯ Geen
- ◯ Heel licht
- ◯ Licht
- ◯ Nogal
- ◯ Ernstig
- ◯ Heel ernstig

In welke mate heeft pijn u de afgelopen 2 weken belemmerd bij uw normale werkzaamheden (zowel werk buitenshuis als huishoudelijk werk)?

- ◯ Helemaal niet
- ◯ Een klein beetje
- ◯ Nogal
- ◯ Veel
- ◯ Heel eg veel

Deze vragen gaan over hoe u zich de afgelopen 2 weken heeft gevoeld. Wilt u bij elke vraag het antwoord aankruisen dat het beste aansluit bij hoe u zich heeft gevoeld. Hoe vaak gedurende de afgelopen 2 weken:

	Voort-durend	Meestal	Vaak	Soms	Zelden	Nooit
Voelde u zich levenslustig?	◯	◯	◯	◯	◯	◯
Voelde u zich erg zenuwachtig?	◯	◯	◯	◯	◯	◯
Zat u zo erg in de put dat niets u kon opvrolijken?	◯	◯	◯	◯	◯	◯
Voelde u zich kalm en rustig?	◯	◯	◯	◯	◯	◯
Voelde u zich erg energiek?	◯	◯	◯	◯	◯	◯
Voelde u zich neerslachtig en somber?	◯	◯	◯	◯	◯	◯
Voelde u zich uitgeblust?	◯	◯	◯	◯	◯	◯
Voelde u zich gelukkig?	◯	◯	◯	◯	◯	◯
Voelde u zich moe?	◯	◯	◯	◯	◯	◯

Hoe vaak hebben uw lichamelijke gezondheid of emotionele problemen gedurende de afgelopen 2 weken uw sociale activiteiten (zoals bezoek aan vrienden of naaste familieleden) belemmerd?

◯ Voortdurend
◯ Meestal
◯ Vaak
◯ Soms
◯ Zelden
◯ Nooit

Wilt u het antwoord kiezen dat het beste weergeeft hoe juist of onjuist u elk van de volgende uitspraken voor uzelf vindt.

	Volkomen juist	Grotendeels juist	Weet ik niet	Grotendeels onjuist	Volkomen onjuist
Ik lijk gemakkelijker ziek te worden dan andere mensen	○	○	○	○	○

	Volkomen juist	Grotendeels juist	Weet ik niet	Grotendeels onjuist	Volkomen onjuist
Ik ben net zo gezond als andere mensen die ik ken	○	○	○	○	○

	Volkomen juist	Grotendeels juist	Weet ik niet	Grotendeels onjuist	Volkomen onjuist
Ik verwacht dat mijn gezondheid achteruit zal gaan	○	○	○	○	○

	Volkomen juist	Grotendeels juist	Weet ik niet	Grotendeels onjuist	Volkomen onjuist
Mijn gezondheid is uitstekend	○	○	○	○	○

Next

Vragenlijsten: Deel 7 van 7

Hieronder vindt u een lijst met woorden. Deze woorden beschrijven stemmingen en gevoelstoestanden. Lees elk woord zorgvuldig en geef aan wat het best weergeeft hoe u zich nu, op dit moment voelt.

Denk niet te lang na over uw antwoord, het gaat om uw eerste indruk. Er bestaan geen foute antwoorden. Elk antwoord is goed, als het maar uw stemming weergeeft.

	Helemaal niet	Een beetje	Enigszins	Nogal	Heel erg
Neerslachtig	○	○	○	○	○
Uitgeput	○	○	○	○	○
Hulpeloos	○	○	○	○	○
Droevig	○	○	○	○	○
Vermoeid	○	○	○	○	○
Eenzaam	○	○	○	○	○
Aan het eind van mijn krachten	○	○	○	○	○
Ongelukkig	○	○	○	○	○
Lusteloos	○	○	○	○	○
Onwaardig	○	○	○	○	○
Doodop	○	○	○	○	○
Droefgeestig	○	○	○	○	○
Afgemat	○	○	○	○	○

Helemaal niet Een beetje Enigszins Nogal Heel erg

Wanhopig ○ ○ ○ ○ ○

Dit waren alle vragenlijsten.

Nu volgt nog het computer spel!

Next
